# Implementation processes and capacity-building needs in Ontario maternal-newborn care hospital settings: a cross-sectional survey

**DOI:** 10.1186/s12912-024-02643-z

**Published:** 2025-01-06

**Authors:** Jessica Reszel, Olivia Daub, Sandra I. Dunn, Christine E. Cassidy, Kaamel Hafizi, Marnie Lightfoot, Dahlia Pervez, Ashley Quosdorf, Allison Wood, Ian D. Graham

**Affiliations:** 1 School of Nursing, University of Ottawa, 200 Lees Avenue, Ottawa, ON K1N 6N5 Canada; 2 Clinical Epidemiology Program, Ottawa Hospital Research Institute, 501 Smyth Road, Ottawa, ON K1H 8L6 Canada; 3 Better Outcomes Registry & Network (BORN) Ontario, 401 Smyth Road, Ottawa, ON K1H 8L1 Canada; 4 School of Communication Sciences and Disorders, Western University, 1201 Western Road, London, ON N6G 1H1 Canada; 5 School of Nursing, Dalhousie University, 5869 University Avenue, Halifax, NS B3H 4R2 Canada; 6 IWK Health Centre, 5980 University Avenue, Halifax, NS B3K 6R8 Canada; 7 Women and Children’s Health Network, Orillia Soldiers’ Memorial Hospital, 170 Colborne St W, Orillia, ON L3V 2Z3 Canada; 8 Parent Research Advisor, Ottawa, ON Canada; 9 Neonatal Intensive Care Unit, The Ottawa Hospital, 501 Smyth Road, Ottawa, ON K1H 8L6 Canada; 10 School of Epidemiology and Public Health, University of Ottawa, 600 Peter Morand Crescent, Ottawa, ON K1G 5Z3 Canada

**Keywords:** Nursing, Implementation science, Implementation practice, Maternal-newborn care, Evidence-informed care, Practice changes, Survey

## Abstract

**Background:**

Maternal-newborn care does not always align with the best available evidence. Applying implementation science to change initiatives can help move evidence-informed practices into clinical settings. However, it remains unknown to what extent current implementation practices in maternal-newborn care align with recommendations from implementation science, and how confident nurses, other health professionals, and leaders are completing steps in the implementation process. We aimed to understand Ontario maternal-newborn teams’ (1) approaches to implementing practice changes and the extent to which their implementation processes aligned with an implementation science planned-action framework; and (2) perceptions of importance and confidence completing implementation activities.

**Methods:**

We conducted a cross-sectional survey between September–November 2023. Using purposive sampling, we invited Ontario maternal-newborn nurses, other healthcare professionals, and leaders who had experience participating in or leading implementation projects to complete an online questionnaire. The questionnaire was informed by an implementation science framework, which includes three core phases (identify issue; build solutions; implement, evaluate, sustain). The questions probed respondents’ perceptions of frequency of completion, importance, and confidence for each of the 28 implementation activities. We used descriptive statistics for the closed-ended questions and grouped the written responses into categories.

**Results:**

We received 73 responses from 57 Ontario maternal-newborn hospitals, the majority being nurses in point-of-care and leadership roles. Nearly all respondents agreed that each of the 28 implementation activities were important. Respondents reported always completing a median of 8 out of 28 activities, with the number of activities completed declining from phase 1 through to 3. Most respondents indicated they were somewhat confident completing the implementation activities and agreed their teams would benefit from increasing their knowledge and skills to use an evidence-informed approach to implementing practice changes.

**Conclusions:**

Despite viewing implementation activities as important, many teams are not consistently doing them and lack confidence, particularly in later phases of the implementation process. These findings inform where further capacity-building and supports may be needed to enable maternal-newborn nurses, other healthcare professionals, and leaders to apply implementation science to their change initiatives.

**Supplementary Information:**

The online version contains supplementary material available at 10.1186/s12912-024-02643-z.

## Background

Although often a healthy event, childbirth is the most frequent reason for hospitalization in Canada and the United States [1, 2]. Given the risk for negative short- and long-term consequences for both the pregnant or birthing person and their infant(s), ensuring that high quality and evidence-based care is provided is essential. Improving maternal-newborn care^1^ has therefore become an international priority and there has been a growing focus on the implementation of quality care in these settings [3]. However, evidence-practice gaps remain. For instance, compared to evidence-informed recommendations, there continues to be overuse of practices such as caesarean births, inductions, and formula supplementation [4], among others. This overuse may further strain limited financial and human resources, lead to patient dissatisfaction, and cause physical, psychological, and social harm [5, 6].

Closing these evidence-practice gaps requires evidence-based *implementation* [7]. Implementation science aims to develop and test methods and strategies to effectively move evidence into practice [8]. Despite a growing body of literature, implementation science has not been well translated into practice-based settings [9]. This has created a “secondary gap” where healthcare teams can identify the need to implement evidence into practice to improve care and outcomes, but they do not necessarily use evidence to inform the processes they use to implement that evidence into practice [10]. However, there are a growing number of examples of teams successfully applying implementation science informed approaches in maternal-newborn care [11–13] and other settings [14], illustrating the potential of implementation science in improving care processes and outcomes in healthcare settings.

Nurses are frequently tasked with leading and supporting change initiatives, and there are increasing calls for nursing to embrace and apply implementation science in their practice to further improve evidence-based practice [10, 15]. Yet many healthcare providers, including nurses, are not aware of or confident using current evidence on how to implement evidence-based programs, guidelines, or innovations [16]. Nurses and other healthcare professionals are often not exposed to implementation science in their training, and clinical demands, lack of resources and time, and lack of professional development opportunities may preclude them from learning about implementation science and its applications to their work [9, 10, 17].

Given that capacity for implementation (i.e., whether you have enough people with the right skills), has been identified as one of the key factors for implementation success [18], understanding current implementation practice and implementation capacity-building needs in nursing and healthcare settings is critical to inform future interventions and supports. As a first step to explore this, our research team previously conducted a secondary qualitative analysis of 22 interviews with nursing managers and directors in Ontario, Canada [19], and compared their described implementation approach to an implementation science framework [20]. Overall, there was variability in implementation steps taken across hospitals. Several implementation steps were described infrequently or sub-optimally, suggesting that real-world processes were not as comprehensive as the implementation framework used for comparison [19]. However, limitations of the secondary analysis were the inability to assess the implementation process at a more detailed activity level, and the lack of information about healthcare providers’ perceptions of the importance or value of different steps and their confidence completing them. To build on this work, we sought to collect more comprehensive and recent primary data from a broader range of Ontario maternal-newborn nurses, other healthcare professionals, and leaders.

### Guiding framework

The implementation framework that guided this study was the Implementation Roadmap [20]. The Implementation Roadmap is a planned-action framework (also known as an implementation process model [21]) that is informed by best practices in implementation science and practice [20]. We chose the Implementation Roadmap as it consolidates concepts and steps from established knowledge translation and implementation frameworks (e.g., Knowledge-to-Action framework [22], CAN-Implement [23]) as well as experiential knowledge from implementation science and practice experts. The Implementation Roadmap was designed to guide professionals, including nurses, through the core phases, steps, and activities of the implementation process (based on best practices in implementation science and practice), making it a suitable framework for exploring the current implementation practices of maternal-newborn teams.

### Study aims

In this study we aimed to understand Ontario maternal-newborn teams’: (1) approaches to implementing practice changes, including who is involved and which activities are done, and how closely their processes align with an implementation science planned-action framework (the Implementation Roadmap [20]); and (2) perceived importance and confidence completing different implementation activities.

## Methods

We used the Checklist for Reporting Of Survey Studies (CROSS) to guide the writing of this manuscript [24] (see Additional file 1 for completed checklist).

### Design

We used a cross-sectional survey design. This study was exploratory in nature and as such, we did not have pre-existing hypotheses for statistical testing.

### Study setting and sampling

The setting for our study was Ontario, Canada, which has over 140,000 births per year. There are 93 hospitals in Ontario that provide perinatal, birthing and newborn care [25], with most of these of births occurring in hospital [26]. Data on nearly every birth is captured in Ontario’s prescribed birth registry, the Better Outcomes Registry & Network (BORN) Ontario, which provides important data on maternal-newborn healthcare and outcomes to contributing hospitals, researchers, and policymakers [27].

We used purposive sampling. Using BORN Ontario’s internal contact list (which included BORN’s primary contacts from 91 out of 93 hospitals), we compiled a list of individuals who were expected to be involved in implementation projects to improve practice, such as directors, nursing managers, clinical nurse educators, and nurses (*N* = 278 contacts across 91 hospitals). While it was anticipated that these contacts would meet the eligibility criteria, if they did not meet eligibility criteria or were unable to participate, we asked them to forward the invitation email to other colleagues (i.e., snowball sampling). We included nearly all Ontario maternal-newborn hospitals in our sampling frame (*n* = 91) and aimed to collect at least one response from each hospital. To assess the representativeness of the sample, we compared the acuity levels and geographical locations of the responding organizations to the distribution across the province as a whole (Additional file 3).

We followed Dillman’s guidelines for survey distribution and reminders [28, 29], with recruitment occurring over a nine-week period (September to November 2023). Contacts received a “pre-notice” email from their designated BORN coordinator, providing an alert about the upcoming questionnaire from a known contact. The lead researcher then sent the survey email invitation and two email reminders (at 2 weeks and 4 weeks) to all contacts. Lastly, the internal BORN coordinators did a final personal telephone or email reminder (as per their usual communication methods) to their non-responding contacts.

### Inclusion and exclusion criteria

The inclusion criteria were individuals who: (1) work in an Ontario maternal-newborn hospital unit (i.e., labour and delivery, postpartum, neonatal intensive care unit, or special care nursery); (2) are responsible for leading, supervising, or participating in implementation projects, quality improvement projects, or practice change initiatives in their current role; and (3) read and understand English.

### Instrument

Because there was no existing validated questionnaire that met our needs, the research team developed the questionnaire, informed by two main sources.

First, we used the Implementation Roadmap activities, which are spread across three main phases: phase 1 involves issue identification and clarification; phase 2 involves building solutions and field-testing them; and phase 3 involves implementing, evaluating, and sustaining [20]. The activities in this framework became a series of 28 questionnaire items to understand which are viewed as important, which are typically done, and which activities respondents feel confident completing (Table 1). We slightly modified the breakdown and wording of the Implementation Roadmap activities to better align with language used by nursing managers and directors in our previous interviews with a similar sample [19] and to increase understandability based on team feedback. The developer of the framework was involved in this process and ensured the integrity of the framework's activities was maintained.

**Table 1:** Implementation Roadmap activities as presented in questionnaire

**PHASE 1 – Issue identification and clarification** 1. Identify a relevant problem or issue 2. Form a working group 3. Involve stakeholders as partners throughout change initiative 4. Create a formal implementation plan 5. Use research evidence to identify potential programs, guidelines, practices, or innovations to solve problem 6. Assess the quality of the program, guideline, practice, or innovation 7. Identify or create a tangible indicator of best practice 8. Collect local data to learn about current practice in your setting 9. Compare current practice in your setting to the best practice to determine how big the “gap” is	
**PHASE 2 – Build solutions and field test them** 10. Work as a team to select the best practice to be implemented 11. Analyze best practice for who needs to do what, when, to whom, and under what circumstances 12. Confirm that key stakeholders endorse the selected best practice 13. Customize the selected best practice for your setting 14. Conduct a stakeholder analysis 15. Assess the barriers and drivers to implementing the best practice 16. Prioritize the identified barriers that are feasible to address 17. Select change strategies to address the identified barriers 18. Field-test the selected change strategies 19. Create a plan for a process evaluation 20. Create a plan for an outcome evaluation	
**PHASE 3 – Implement, evaluate, sustain** 21. Complete a pre-launch checklist 22. Create a sustainability plan 23. Use data to assess if the best practice is being used 24. Use data to assess if use of best practice resulted in the desired outcomes 25. Use the monitoring and evaluation findings to adjust the change strategies 26. Use strategies to sustain use of the best practice over time 27. Use data to assess if sustainability strategies are maintaining use of best practice	
**Throughout implementation process** 28. Consider equity, diversity, and inclusion (EDI)	

Second, we used our findings from a previous secondary qualitative analysis of interviews with maternal-newborn nursing leaders [19] to identify areas for exploration in this current survey. Our previous study identified the need to better understand the specific roles involved in the various implementation phases, the mechanisms for engagement, and considerations for equity, diversity, and inclusion (EDI); we included these topics in the questionnaire used in this study to probe these areas.

The research team, which included experts in nursing, implementation science, and implementation practice, reviewed the study questionnaire to assess its face validity. The questionnaire was piloted by two maternal-newborn nurses with experience leading implementation initiatives. Changes were made to enhance clarity (e.g., defining terms, including examples), flow (e.g., re-ordering the questions), and functionality (e.g., changing large matrices to individual questions). While feedback suggested the questionnaire was long and may not be feasible for busy healthcare providers and leaders to complete, we made the decision to maintain the length of the questionnaire to facilitate a comprehensive understanding of respondents’ views and approaches to implementing practice changes.

The final questionnaire had 159 questions across six parts. Question formats included 135 closed-ended questions (including multi-select, single-select, and rating scale formats) and 24 open-ended questions (including substitution, extension, and expansion questions [30]). This paper reports on the first three questionnaire sections: eligibility screening, demographics, and implementation process and steps (see Additional file 2 for full questionnaire).

### Data collection

Respondents were asked to complete an online questionnaire hosted in REDCap (Research Electronic Data Capture), a secure, web-based software platform designed to support data capture for research studies [31]. The questionnaire took approximately 20 to 30 min to complete. The questionnaire was an “open survey,” meaning there was one generic link for all respondents. To minimize the risk of multiple entries by the same person, we collected detailed demographic information (including hospital name, unit name, and role), which allowed us to identify and remove duplicate entries if needed.

### Data analysis

We imported the questionnaire responses into SPSS (version 29) for analysis. We used descriptive statistics for the close-ended questions. We excluded records that did not answer any questions beyond the 28 Implementation Roadmap activities. For nominal and ordinal data, we present frequencies and percentages to show the response distribution across categories. For continuous data, we present measures of central tendency (medians, interquartile ranges [IQR], ranges). For open-ended text boxes, we grouped the written responses into categories and provide frequency counts, where appropriate, to illustrate recurring topics. There was some missing data from respondents skipping questions; we indicate the denominator throughout to make clear the number of respondents and missingness for each question.

### Ethical considerations

This study was approved by the Children’s Hospital of Eastern Ontario (CHEO) Research Ethics Board (protocol #23/59X) and the University of Ottawa Research Ethics Board (H–12–23–9959)*.* The questionnaire homepage included detailed information on the study and consent was implied by starting the questionnaire. The questionnaire included indirect identifiers (e.g., hospital name, unit type, role), which together could identify the respondent; however, these were optional. To protect confidentiality, only the required research team members had access to the REDCap database and identifying information was saved in a master list separate from the data.

## Results

**Fig. 1:**
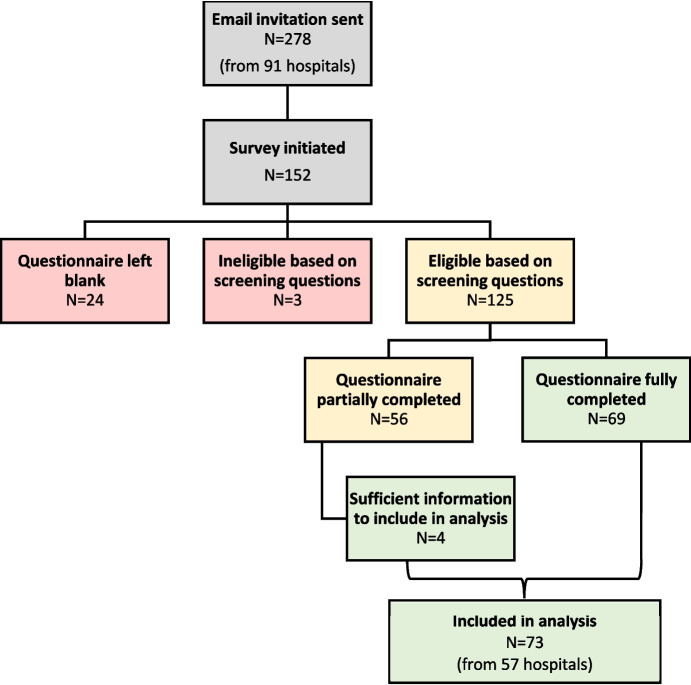
Response flow diagram. Note: We calculated the individual response rate by dividing the number of questionnaires included in the analysis (*n* = 73 questionnaires) by the number of people who received the questionnaire (*n* = 278 people) (73/278, individual response rate = 26%). We calculated the organizational response rate by dividing the number of maternal-newborn hospitals with at least one response (*n* = 57 hospitals) by the number of maternal-newborn hospitals that were contacted (*n* = 91 hospitals) (57/91, organizational response rate = 63%). We calculated the questionnaire completion rate by dividing the number of questionnaires in the analysis (*n* = 73) by the number of questionnaires initiated by eligible individuals (*n* = 125 people started the questionnaire) (73/125, questionnaire completion rate = 58%)

Between September 20, 2023 and November 22, 2023, 73 individuals completed the questionnaire. Figure 1 presents a flow diagram of the number of responses received and those that were included in this analysis. We obtained a response rate of 26% (73/278). Across the 73 respondents, there was at least one response from nearly two-thirds of Ontario maternal-newborn hospitals (57/91, 63% of maternal-newborn hospitals). There were 13 hospitals that had a response from more than one person; we included these records as the individuals represented different units and/or different professional roles.

### Demographic and contextual information

Respondents came from a variety of roles and settings (Table 2), with the majority being nurses working in various clinical and leadership capacities (*n* = 58, 80.6%). Most respondents identified as women, which is consistent with the distribution of the overall nursing workforce in the geographical region where this survey was conducted [32]. The distribution of geographical locations and acuity levels of the 57 hospitals respondents were from were very similar to that of all maternal-newborn hospitals in the province (see Additional file 3 for comparison). Thirty respondents (30/73, 41%) indicated they had training in implementation: 10 reported training in implementation science (10/73, 14%) and 29 reported training in knowledge translation/implementation practice (29/73, 40%).

**Table 2:** Respondents’ demographic information

**Characteristic**	
**Gender identity (*N* = 73) – n (%)**	
Woman	71 (97.3)
Man	1 (1.4)
Prefer not to answer	1 (1.4)
**Professional role (*N* = 72) – n (%)**	
Manager	32 (44.4)
Nurse	12 (16.7)
Nurse educator	7 (9.7)
Program director	5 (6.9)
Midwife	5 (6.9)
Quality improvement or implementation support	3 (4.2)
Advanced practice nurse	2 (2.8)
Physician	1 (1.4)
Other (care facilitator; charge nurse; clinical lead)	5 (6.9)
**Highest level of education completed (*N* = 71) – n (%)**	
College diploma	14 (19.7)
Bachelor’s degree	38 (53.5)
Master’s degree	18 (25.4)
Doctoral degree	1 (1.4)
**Type of maternal-newborn unit respondent works in (*N* = 73) – n (%)**^**a**^	
Labour and delivery unit	59 (80.8)
Postpartum unit	53 (72.6)
Neonatal intensive care unit or Special care nursery	25 (34.2)
**Level of acuity**^**b**^** (*N* = 72) – n (%)**	
Low risk (level 1)	27 (37.5)
Moderate risk (level 2)	30 (41.7)
High risk (level 3)	15 (20.8)
**Geographical region**^**b**^** (*N* = 72) – n (%)**	
Central Ontario	20 (27.8)
East Ontario	17 (23.6)
North East Ontario	8 (11.1)
North West Ontario	6 (8.3)
Toronto Ontario	6 (8.3)
West Ontario	15 (20.8)
**Years of experience in maternal-newborn care (*N* = 73)**	
Median	17
Interquartile range	12
Range	0–35
**Years of experience *supervising or leading* practice change initiatives or implementation/quality improvement projects (*N* = 61)**^**c**^	
Median	5
Interquartile range	8
Range	1–29
**Years of experience *participating in* practice change initiatives or implementation/quality improvement projects (*N* = 47)**^**d**^	
Median	10
Interquartile range	10
Range	1–30

^a^Respondents could select more than one response option

^b^As per the Provincial Council for Maternal and Child Health (PCMCH) [25]

^c^Only respondents who indicated they had experience leading or supervising practice change initiatives/implementation projects were asked this question

^d^Only respondents who indicated they had experience participating in practice change initiatives/implementation projects were asked this question

Overall, respondents identified there was room to improve the quality of care in maternal-newborn settings. Fifty-five respondents (75.3%) felt the proportion of inappropriate care (overuse, underuse, misuse) in Ontario maternal-newborn care settings was about the same as the 30% median reported in a recent national study [33]. Thirteen respondents (17.8%) thought the proportion was higher, reporting a median of 50% (range = 38–74%). Five respondents (6.8%) thought the proportion was lower, reporting a median of 20% (range = 20–25%).

Respondents (*n* = 69) wrote examples of clinical practice changes they are currently implementing or are planning to implement in the future (see Additional file 4 for full list of categories). The most common topics included infant feeding practices (breastfeeding) (*n* = 25, 36%), inductions (*n* = 22, 32%), general education and training initiatives (*n* = 18, 26%), caesarean births (*n* = 16, 23%), internal service re-organization (*n* = 16, 23%), and oxytocin (*n* = 13, 19%).

### Implementation process and activities

We present our findings on approaches to implementing practice changes in three main categories: (1) current use of any formal implementation processes and frameworks; (2) individuals involved in implementation efforts, organized by the three Implementation Roadmap phases; and (3) perceived importance and completion of implementation activities, organized by the 28 activities in Implementation Roadmap.

#### Current use of any formal implementation processes and frameworks

Just over half of respondents reported that their organization either did not have a formal process to facilitate practice changes (*n* = 18/69, 26.1%) or they were unsure whether it had a process (*n* = 17/69, 24.6%). The remaining respondents indicated that their organization had a mandatory process (*n* = 16/69, 23.2%) or an optional process (*n* = 18/69, 26.1%) to facilitate practice changes.

For those that answered they had a mandatory or optional process, respondents briefly described their process. Of the 25 that answered, 11 (44%) identified an approach that typically guides their change process (e.g., continuous quality improvement). Thirteen respondents (52%) described their process as using different committees, meetings, and approval processes. Seventeen respondents also indicated that they used a formal framework, model, or theory to facilitate the change process; the most frequent examples were Plan, Do, Study, Act (PDSA) (*n* = 13), root cause analysis (*n* = 13), and Lean methodology (*n* = 10).

#### Individuals involved in hospital implementation efforts

Respondents reported different roles that are typically involved in the core phases of the Implementation Roadmap (Table 3). Nurse managers and clinical nurse educators were most frequently responsible for leading the process. Nurses at point-of-care, physicians, and nurse managers were most frequently involved in identifying the problem to be addressed; nurse managers and clinical nurse educators were most frequently involved in developing the solutions; and clinical nurse educators, nurse managers, and nurses at point-of-care were most frequently involved in implementing the solutions. The number of roles involved varied by phase, slightly decreasing as teams worked through the implementation phases.

**Table 3:** Roles and involvement in core phases of implementation process (*N* = 70)

	**n (%)**			
	**Primarily responsible for *leading* the overall implementation process**	***Identifying a problem* that needs to be addressed**	***Developing solution(s)* to address the problem**	***Implementing* the *solution(s)* in practice**
Program director – n (%)	21 (30.0)	39 (55.7)	32 (45.7)	16 (22.9)
Manager – n (%)	50 (71.4)	58 (82.9)	59 (84.3)	53 (75.7)
Clinical educator – n (%)	47 (67.1)	46 (65.7)	57 (81.4)	58 (82.9)
Nurses working at point-of-care – n (%)	24 (34.3)	63 (90.0)	50 (71.4)	53 (75.7)
Physicians – n (%)	30 (42.9)	61 (87.1)	45 (64.3)	38 (54.3)
Midwives – n (%)	14 (20.0)	45 (61.6)	37 (52.9)	33 (47.1)
Decision support personnel/analysts/QI – n (%)	5 (7.1)	13 (18.6)	13 (18.6)	7 (10.0)
Pregnant and birthing people – n (%)	5 (7.1)	40 (57.1)	18 (25.7)	4 (5.7)
Family members/caregivers – n (%)	5 (7.1)	40 (57.1)	20 (28.6)	7 (10.0)
Median number of roles involved – median (IQR)	2 (1.25)	6 (4)	5 (3)	4 (3)

Respondents (*N* = 70) identified various systems for engaging people in the implementation process including staff huddles (*n* = 64, 91.4%), patient and family advisory councils (*n* = 44, 62.9%), staff unit councils (*n* = 37, 52.9%), ticket systems for staff to submit ideas and feedback (*n* = 33, 47.1%), and ticket systems for patients and families to submit ideas and feedback (*n* = 32, 45.7%).

#### Importance and completion of implementation activities

**Fig. 2:**
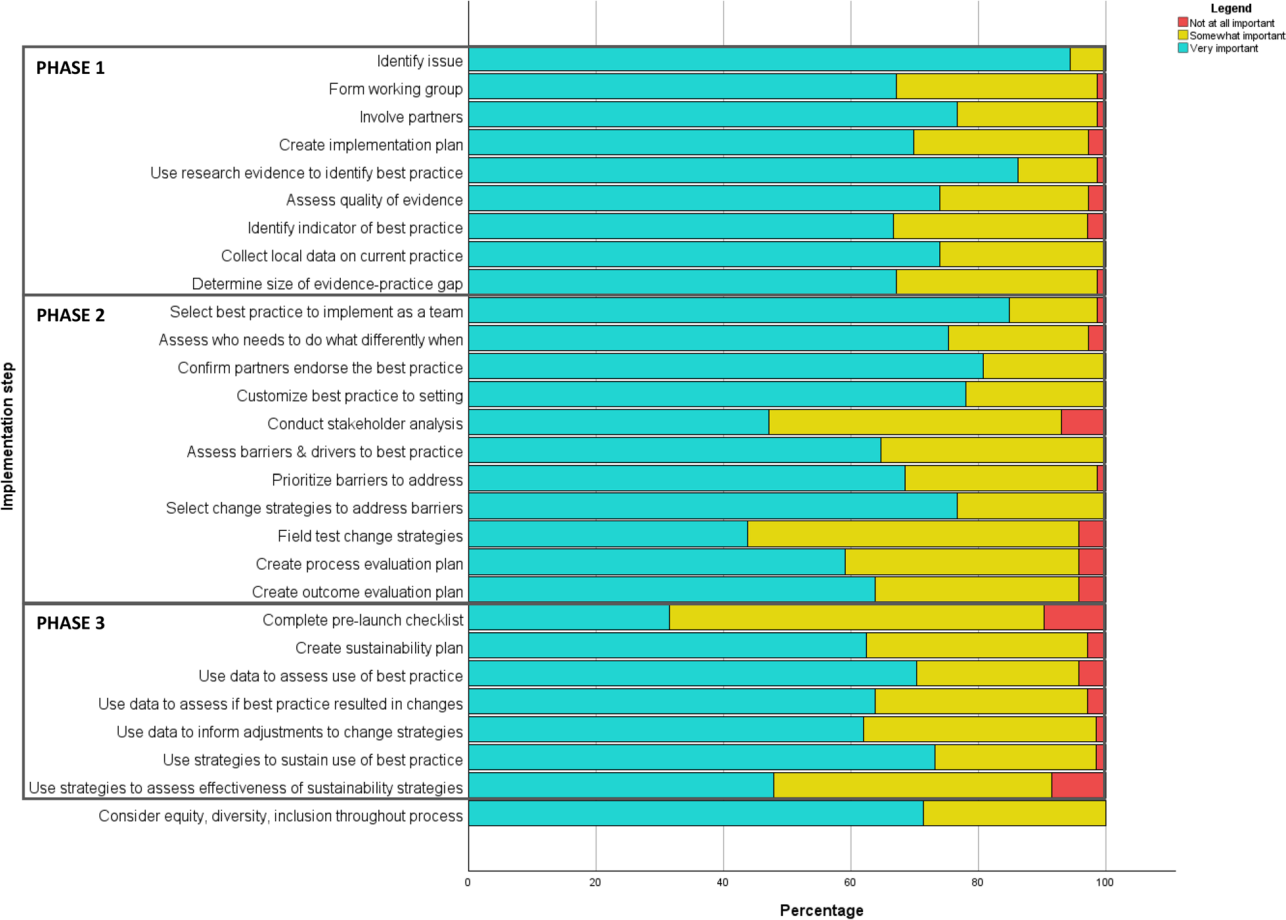
Perceptions of importance of each implementation activity

Nearly all respondents (> 90%) agreed that each of the 28 activities in the implementation process were “very” or “somewhat” important (Fig. 2).

Nearly half of respondents indicated their teams “always” or “sometimes” complete each of the 28 implementation activities (*n* = 29/63, 46%). Respondents reported their teams “always” complete a median of 8 out of 28 activities (29% of activities, range = 0–28). The number of activities reported as “always” completed declined from phase 1 through to 3: respondents “always” completed a median of 4 of 9 activities in phase 1 (44% of phase 1 activities), a median of 3 of 11 activities in phase 2 (27% of phase 2 activities), and a median of 0 of 7 activities in phase 3 (0% of phase 3 activities). Additional file 5 illustrates the different implementation activities reported by each respondent with complete data (*n* = 63).

**Fig. 3:**
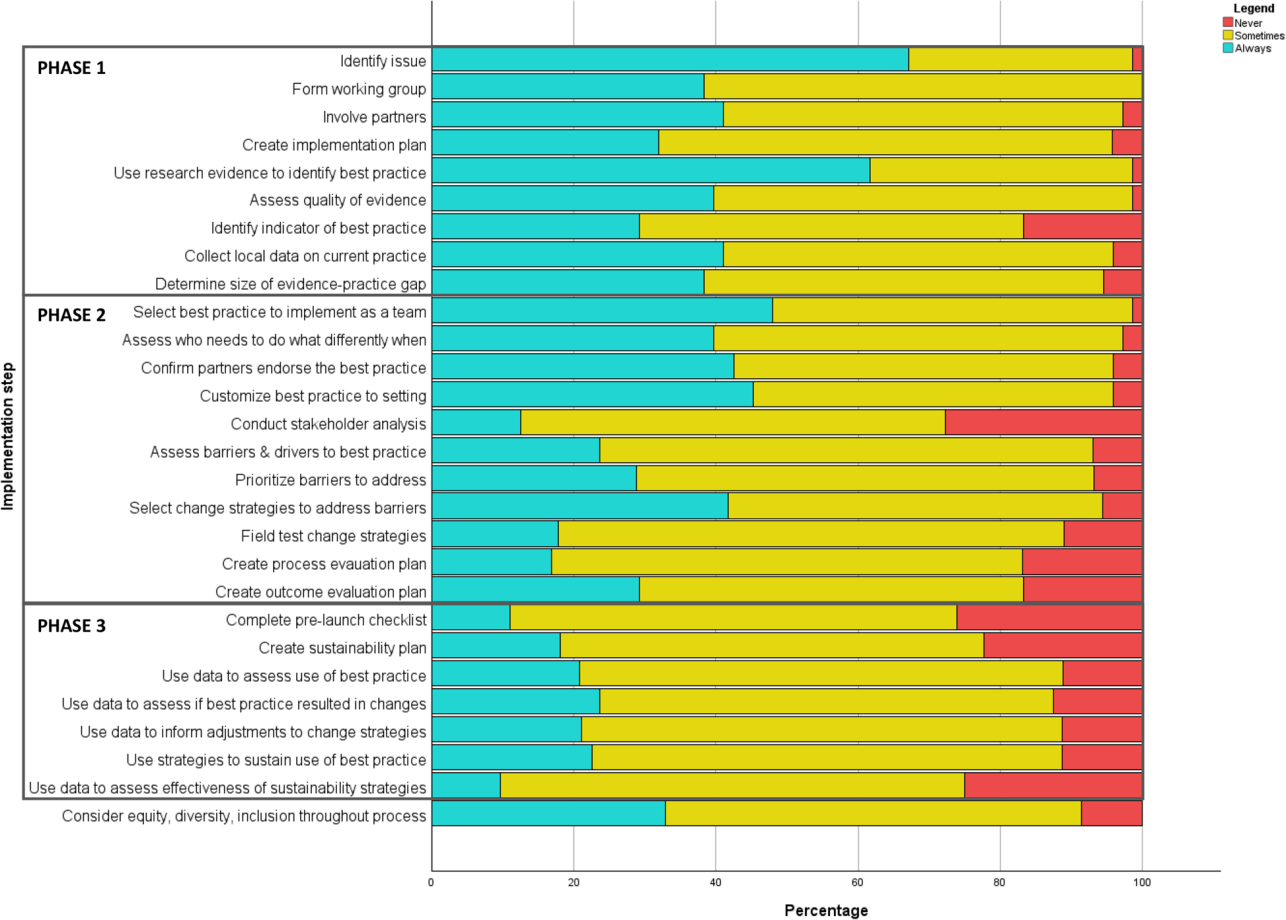
Perceptions of how often each implementation activity is done

Figure 3 illustrates respondents' perceptions of how frequently the 28 implementation activities are completed in their settings. The seven activities (i.e., bottom quartile) that were most frequently reported as “never” done were: conduct a stakeholder analysis (*n* = 20/72; 27.8%); complete a pre-launch checklist (*n* = 19/73; 26.0%); use data to assess if sustainability strategies are maintaining use of best practice (*n* = 18/72, 25.0%); create a sustainability plan (*n* = 16/22, 22.2%); create a plan for a process evaluation (*n* = 12/71, 16.9%); identify or create a tangible indicator of best practice (*n* = 12/72, 16.7%); and create a plan for an outcome evaluation (*n* = 12/72, 16.7%). Six of these seven activities were from phase 2 or 3 of the implementation process. The complete data (frequencies and percentages) by implementation activity are presented in Additional file 6.

When asked if equity, diversity, and inclusion (EDI) are considered in the implementation process, 23 (32.9%) reported “always,” 41 (58.6%) reported “sometimes,” and 6 (8.6%) reported “never” considering EDI. Of those who stated they “always” or “sometimes” consider EDI, the steps that most frequently included EDI considerations were consulting with interested and affected parties (*n* = 46/64, 71.9%) and when learning about potential barriers to implementing solutions (*n* = 44/64, 68.8%) (see Additional file 7).

When comparing the frequencies across implementation activities that were rated as “very” important and also reported as “always” done, we observed the most congruence in phase 1, and the least congruence in phase 3 (Table 4). For example, of the 69 respondents who indicated that identifying a relevant problem or issue is “very important,” 49 (71%) said they “always” did the activity and 20 (29%) reported only doing it “sometimes” or “never.”

**Table 4:** Proportion of respondents reporting their teams view an activity as “very important” who also said their team “always” does it

	**Implementation activity**	**n/N (%)**^**a**^
**Phase 1**	Identify a relevant problem or issue	**49/69 (71.0)**^**b**^
	Form a working group	26/49 (53.1)
	Involve stakeholders as partners throughout change initiative	30/56 (53.6)
	Create a formal implementation plan	23/51 (45.1)
	Use research evidence to identify potential programs, guidelines, practices, or innovations to solve problem	**44/63 (69.8)**^**b**^
	Assess the quality of the program, guideline, practice, or innovation	**29/54 (53.7)**^**b**^
	Identify or create a tangible indicator of best practice	21/48 (43.8)
	Collect local data to learn about current practice in your setting	**29/54 (53.7)**^**b**^
	Compare current practice in your setting to the best practice to determine how big the “gap” is	**28/49 (57.1)**^**b**^
**Phase 2**	Work as a team to select the best practice to be implemented	**35/62 (56.5)**^**b**^
	Analyze best practice for who needs to do what, when, to whom, and under what circumstances	28/55 (50.9)
	Confirm that key stakeholders endorse the selected best practice	31/59 (52.5)
	Customize the selected best practice for your setting	**31/57 (54.4)**^**b**^
	Conduct a stakeholder analysis	**9/33 (27.3)**^**c**^
	Assess the barriers and drivers to implementing the best practice	16/46 (34.8)
	Prioritize the identified barriers that are feasible to address	21/50 (42.0)
	Select change strategies to address the identified barriers	30/56 (53.6)
	Field-test the selected change strategies	13/32 (40.6)
	Create a plan for a “process evaluation”	**12/42 (28.6)**^**c**^
	Create a plan for an “outcome evaluation”	21/46 (45.7)
**Phase 3**	Complete a pre-launch checklist	8/23 (34.8)
	Create a “sustainability plan”	**13/45 (28.9)**^**c**^
	Use data to assess if the best practice is being used	**15/50 (30.0)**^**c**^
	Use data to assess if use of best practice resulted in the desired outcomes	17/46 (37.0)
	Use the monitoring and evaluation findings to adjust the change strategies	**15/44 (34.1)**^**c**^
	Use strategies to sustain use of the best practice over time	**16/52 (30.8)**^**c**^
	Use data to assess if sustainability strategies are maintaining use of best practice	**7/34 (20.6)**^**c**^
	Consider equity, diversity, and inclusion (EDI) throughout the implementation process	22/50 (44.0)

^a^The denominator (N) is the number of respondents who reported the activity is “very important.” The numerator (n) includes respondents who reported the activity is “very” important AND reported they “always” did it

^b^Top quartile

^c^Bottom quartile

Overall, respondents who indicated their organization had a mandatory or optional formal process or framework to facilitate practice changes reported completing more implementation activities than those who did not have a formal process or framework (Table 5).

**Table 5:** Comparison of number of implementation activities “always” done and use of formal processes and frameworks (*n* = 62)

	**Number of 28 implementation activities “always” done**^**a**^	
	**Median (IQR)**	**Range**
Organization has *mandatory* formal process to facilitate change process (*n* = 15)	9 (13)	3–26
Organization has *optional* formal process to facilitate change process (*n* = 14)	8.5 (20.25)	0–28
Organization *does no*t have formal process to facilitate change process (*n* = 16)	5 (6.5)	0–21
*Unsure* if organization has formal process to facilitate change process (*n* = 17)	9 (7.5)	0–19
***Of those who said they used a “mandatory” or “optional” formal process to facilitate the change process:***		
Use a formal framework, model, or theory to facilitate change process (*N* = 14^b^)	15 (13.25)	5–28
Do not use formal framework, model, or theory to facilitate change process (*N* = 10^b^)	4.5 (10.75)	0–26

^a^Only includes respondents with complete data on all implementation activities

^b^Only includes those who said they used a “mandatory” or “optional” formal process to facilitate the change process

### Perceived confidence and implementation capacity-building needs

About two-thirds of respondents reported receiving formal training in one or more of quality improvement (*n* = 45/73, 61.6%), knowledge translation and implementation practice (*n* = 29/73, 39.7%), and implementation science (*n* = 10/73, 13.7%). Respondents most frequently rated their knowledge and confidence in using evidence-informed approaches to change clinical practice as “moderate.” Those who reported having previous formal training more frequently reported higher levels of knowledge and confidence (see Additional file 8).

**Fig. 4:**
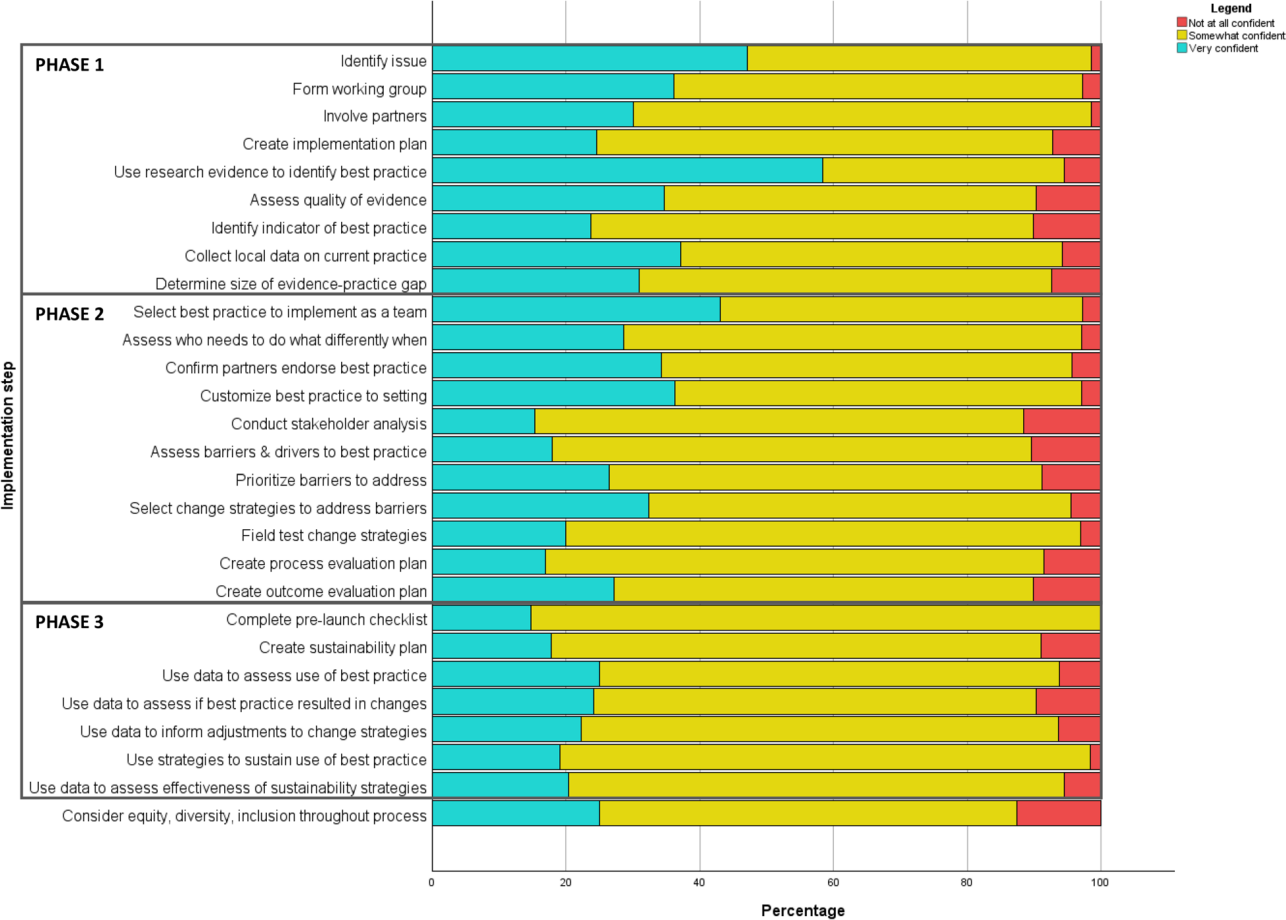
Perceptions of confidence doing each implementation activity. Note: Only respondents who indicated they “always” or “sometimes” do the step were asked about their confidence levels

Figure 4 illustrates respondents’ perceptions of their teams’ confidence for each of the 28 implementation activities. Most respondents indicated their teams were “somewhat” confident across the activities, except for “use research evidence to identify best practice,” where the majority said “very” confident. There were several activities where more than 10% of the sample indicated their teams were “not at all” confident: consider equity, diversity, and inclusion (EDI) (12.5%), conduct a stakeholder analysis (11.5%), assess barriers and drivers to implementing the best practice (10.4%), identify or create a tangible indicator of best practice (10.2%), and create a plan for an outcome evaluation (10.2%).

Overall, most respondents strongly agreed or agreed that their teams would benefit from increasing their knowledge (88.4%) and skills (92.8%) on how to use an evidence-informed approach to implementing clinical practice changes. In addition, most respondents strongly agreed or agreed that their team could benefit from having an evidence-informed process (89.8%) and would value resources to guide the implementation process (95.7%) (Table 6).

**Table 6:** Respondent perceptions of needs related to evidence-informed approaches for implementing practice changes (*N* = 69)

	**n (%)**				
	**Strongly agree**	**Agree**	**Neither agree nor disagree**	**Disagree**	**Strongly disagree**
Our team could benefit from *increasing our knowledge* about evidence-informed approaches to implementing clinical practice changes	30 (43.5)	31 (44.9)	6 (8.7)	2 (2.9)	0 (0)
Our team could benefit from *increasing our skills* to apply evidence-informed approaches to implementing clinical practice changes	32 (46.4)	32 (46.4)	4 (5.8)	1 (1.4)	0 (0)
Our team could benefit from *having an evidence-informed process* to guide the clinical practice change process in our setting	29 (42.0)	33 (47.8)	6 (8.7)	1 (1.4)	0 (0)
Our team is motivated to *use evidence-informed processes and strategies* to bring about clinical practice changes	29 (43.3)	28 (41.8)	10 (14.9)	0 (0)	0 (0)
Our team would value (appreciate) a *resource or toolkit* to guide our clinical practice change process	44 (63.8)	22 (31.9)	2 (2.9)	1 (1.4)	0 (0)

## Discussion

In this study we aimed to explore the implementation processes and implementation capacity-building needs of maternal-newborn nurses, other healthcare professionals, and leaders. We grounded our data collection in an implementation planned-action framework, the Implementation Roadmap, to identify how usual implementation practices compare to recommendations from implementation science. We also learned about respondents’ perceptions of importance and confidence completing various implementation activities. Together, this highlights specific phases and activities where maternal-newborn teams may need additional support in the implementation process.

About half of respondents indicated their organization had a mandatory or optional practice change process or framework, and we observed that these respondents more frequently reported always completing activities in the Implementation Roadmap. This is unsurprising given that theories, models, and frameworks encourage a systematic and structured implementation process and creates a shared language and understanding among team members, which may ultimately increase success and sustainability [10, 16, 34]. In addition, some respondents named a formal theory, model, or framework they use. Similar to our previous findings [19], most of these were approaches and tools grounded in quality improvement rather than implementation planned-action frameworks. With the growing recognition that implementation science can inform and improve change initiatives in healthcare settings, it will be important to consider how implementation science and quality improvement practice can work together to achieve optimal processes and outcomes [35].

Respondents identified a variety of roles that are involved in different phases of the implementation process. Nurses in both leadership and clinical roles were the most frequently selected role for leading change initiatives, identifying problems to address, developing solutions, and implementing solutions. These results highlight the substantial and essential role that nurses play in implementing practice changes [15].

We observed that overall, there was strong involvement from nursing managers throughout the implementation process, which is promising given that leadership behaviors of nurse managers affect the unit climate for the implementation of evidence-based practices [36]. However, the involvement of senior leadership was lower, which likely reflects their role in providing general support and infrastructure rather than engaging in the day-to-day activities. Senior nurse leaders have been identified as a key group that can embed implementation science in their organizations [37]. Given our findings that evaluation and sustainability planning and execution were some of the least frequently completed activities, there may be a particularly important role for senior nurse leaders to play in supporting these activities.

We observed that pregnant and birthing people and their families were less frequently involved in the implementation process, a finding consistent with our previous qualitative study [19]. While about half of respondents indicated pregnant and birthing people and their families were involved in identifying problems (likely through patient feedback systems and patient and family advisory committees), there was a notable decrease in the number of respondents who said they also involved them in developing and implementing solutions. This may be a missed opportunity given that involving patients in co-designing solutions can result in improvements to care, service delivery, and governance [38].

Overall, we found that most respondents perceived the three phases and 28 implementation activities to be important, aligning with our previous work in a different setting [39]. Despite these generally positive ratings of importance, we observed that these implementation activities were not always done. There are several potential explanations for the discrepancy between viewing an activity as important but not doing it. Implementation is complex and there are multi-level barriers to implementing evidence in practice [40]. Implementation is often done with limited time and resources in conditions that are rapidly changing, resistant to change, and subject to policies and regulations [41], and this may account for some implementation activities not being feasible to complete, despite being viewed as important. It is also possible that the difference relates to a lack of knowledge and skill in how to complete the activity. This is consistent with respondents most frequently indicating they were “somewhat” confident across the implementation activities, suggesting that room for improvement exists. Given that healthcare professionals are typically not taught about implementation and improvement practice in their training, nor are there sufficient professional development opportunities in this area [9, 42], it is unsurprising that nurses and other healthcare professionals are reported to have low knowledge [43] and skill proficiency in quality improvement [44] and low confidence in knowledge translation and implementation [45, 46].

We observed a general decrease in respondents’ ratings of importance, completion, and confidence when moving across the three implementation phases. Variability in practitioner perceptions across the implementation process has been reported in previous studies [45–48]. For instance, allied health professionals reported higher confidence levels in the earlier steps of the implementation process (e.g., identifying an evidence-practice gap, finding and appraising evidence), and reported lower confidence levels with activities later in the process (e.g., implementation, monitoring, evaluation) [45, 46]. McNett et al. [48] described three core implementation stages (initiating, maintaining, completing and sustaining) and found that respondents reported moderate difficulty completing each of the stages, with the difficulty ratings increasing across stages. Similarly, respondents rated their success higher in the first phase, with success ratings decreasing in stages 2 and 3. This aligns with our findings that respondents’ ratings of importance, completion, and confidence were highest in the first phase, and decreased in later phases, and signals that nurses, other healthcare professionals, and leaders may need additional support in later implementation activities.

Most respondents identified a need to increase their knowledge and skills in using an evidence-informed approach to implementing practice changes. Our study and previous literature [45, 46] suggest that nurses and other healthcare professionals are in fact interested in learning more about how to implement evidence into practice. Respondents who had previous training in implementation, quality improvement, or knowledge translation rated their knowledge and confidence more positively than those with no training. Implementation capacity-building interventions may result in positive outcomes including increased knowledge and understanding of implementation and an increase in self-reported ability to implement [49]. While we acknowledge that training and tools alone would be insufficient, it is promising to see there is an appetite for further learning about how to effectively move evidence into practice. Ongoing work is needed to develop and evaluate evidence-informed tools, resources, and training initiatives to support the application of implementation science in clinical and health services settings.

### Strengths and limitations

Despite use of strategies known to improve response rates (e.g., pre-notice, reminders) [50], our individual response rate and questionnaire completion rate were only 26% and 58%, respectively. These numbers may reflect the feasibility of the questionnaire for busy healthcare providers and leaders, which took longer than the recommended 10 minutes [50]. Despite this, we obtained at least one response from nearly two-thirds of maternal-hospitals in Ontario, providing good representation of both geographical locations and acuity levels of maternal-newborn units across the province. However, due to recruitment occurring in Ontario, Canada only, the sample may not be representative of maternal-newborn teams in other jurisdictions, and the findings should therefore be generalized with caution.

It is important to note that most respondents were nurses and therefore our findings likely do not reflect the perceptions of other healthcare professionals. However, this nursing perspective is essential given that nurses are often the ones responsible for leading and participating in implementing practice changes. Another limitation is that all data is self-reported; this means that when a respondent said their team completed an implementation activity, we do not know whether this is accurate, nor can we say that it was done optimally. In addition, it is possible that respondents may have answered questions in a way that reflected positively on themselves and their units (i.e., social response bias). However, in many cases, respondents indicated activities their teams did not complete or areas where they were not confident, suggesting that respondents were likely not overestimating.

Finally, it is important to acknowledge that although we assessed face validity, the questionnaire did not undergo validity and reliability testing. In addition, in the questionnaire section with the 28 Implementation Roadmap activities we used a simple three-point scale to assess importance, completion, and confidence. While this decision was made to make the lengthy questionnaire easier to complete, we acknowledge that this poses limitations for our analysis, preventing us from obtaining a more nuanced understanding of respondents’ ratings.

### Recommendations for research

This work highlights several opportunities for future research. First, while this study identified general patterns across implementation phases, we do not know the reasons for these differences. Future research could explore these gaps by asking respondents for more details on the “why” behind these findings. Second, given the limited evidence on how implementation science is being applied by practitioners in healthcare settings, further research is needed to explore this in settings beyond maternal-newborn care and in other jurisdictions. Third, while in this study we focused on describing implementation processes, future research is needed to understand how different implementation steps and activities result in different clinical and implementation outcomes. Finally, promoting equity and better engaging patients throughout the implementation process have been identified as implementation research priorities in maternal health [51]. While we assessed how frequently respondents consider equity and engage patients throughout implementation, further research is needed to develop effective strategies to facilitate these essential components of effective, person-centered, and equitable implementation.

### Implications for nursing

Implementing best practices in organizations is a collective activity, and leadership has been identified as an important mediator that can facilitate or hinder implementation [52]. Nurse managers have indicated a desire to learn about implementation and better support clinical staff [53]; ongoing supports and opportunities are needed to further enhance nursing leaders’ ability to foster environments that facilitate implementation. There are also considerations for nursing education. To date, nursing education programs have embedded evidence-based practice and research methods in curricula; however, there is a need to also include content on how to actually implement evidence into practice [54], including exposure to implementation science and its application to nursing practice.

## Conclusion

While respondents generally viewed implementation activities as important, teams did not always complete the steps or feel confident doing them, particularly in later phases of the implementation process. These findings support the need and interest for further resources, support, and training in using an evidence-informed approach to implementation and will inform the development of a tailored resource for nurses, other healthcare professionals, and leaders in maternal-newborn care settings. Until health systems adopt an evidence-informed process to implement evidence-informed practices, patients will continue to be denied optimal care, resources will be wasted, and equitable health outcomes will be challenging to achieve.

## Supplementary Information

Additional file 1. Checklist for Reporting Of Survey Studies (CROSS). This file includes the 40 CROSS COREQ (Checklist for Reporting Of Survey Studies) items, with corresponding manuscript sections, to indicate where the information is reported.

Additional file 2. Study questionnaire. This file includes the full questionnaire that was administered in this study.

Additional file 3. Organizational-level demographics. This file includes a table presenting characteristics of the respondents’ organizations, including a comparison to the province at large.

Additional file 4. Categories of practice change initiatives/implementation projects identified by survey respondents. This file includes a table presenting the different categories of practice change initiatives and implementation projects respondents indicated their organizations are currently or planning to work on.

Additional file 5. Implementation activities done (reported by respondent). This file includes a figure presenting the responses on frequency of implementation activity completion (always, sometimes, never) for the 63 respondents for whom we had complete data on that series of questions.

Additional file 6. Importance, completion, and confidence with Implementation activities. This file includes a table presenting the descriptive statistics (frequency and percentages) for importance, completion, and confidence for each of the 28 implementation activities.

Additional file 7. Equity, diversity, and inclusion (EDI) considerations during implementation process. This file includes a table presenting data on how respondents indicated they integrated equity, diversity, and inclusion (EDI) considerations into the implementation process.

Additional file 8. Training, knowledge, and confidence related to using an evidence-informed approach to implement clinical practice changes. This file includes a table with the cross tabulation of respondents’ self-rated knowledge and confidence by whether they had previous training in of quality improvement, knowledge translation, implementation practice, or implementation science.

## Data Availability

The data that support the findings of this study are available from the corresponding author upon reasonable request.

## Abbreviations

BFI: Baby Friendly Initiative
BORN: Better Outcomes Registry & Network
EDI: Equity, Diversity, Inclusion
HBHC: Healthy Babies Healthy Children
LDRP: Labor, Delivery, Recovery, and Postpartum
MORE^OB^: Managing Obstetrical Risk Efficiently
NICU: Neonatal Intensive Care Unit
OTAS: Obstetric Triage Acuity Scale
PCMCH: Provincial Council for Maternal and Child Health
PDSA: Plan, Do, Study, Act
PPH: Postpartum Hemorrhage
SOGC: Society of Obstetricians and Gynaecologists of Canada
TOLAC: Trial Of Labor After Caesarean

## Footnotes

1 *In this paper we have used the term “maternal-newborn” to refer to pregnant and birthing people and their infants receiving care in hospital. The term “maternal” is related to the word “mother”; we acknowledge that not every pregnant and birthing person identifies as a mother.*
