# Supplementary material for: Implementation processes and capacity-building needs in Ontario maternal-newborn care hospital settings: a cross-sectional survey
Source: BMC Nurs. 2025 Jan 6;24:10. doi: 10.1186/s12912-024-02643-z (PMC11702017; doi:10.1186/s12912-024-02643-z)
Supplement: Supplementary file 2 — Additional file 2. Study questionnaire. This file includes the full questionnaire that was administered in this study. [file 12912_2024_2643_MOESM2_ESM.docx]

**Additioanl File 2: Study questionnaire**

**Note that this paper reports on data from Parts A, B, C only.*

**Part A: Screening Questions**

1. Are you involved in maternal-newborn care in an Ontario hospital? (e.g., labour and delivery, postpartum, neonatal intensive care)
   - Yes
   - No
2. In your current role, do you:

*[please check all that apply]*

- *Supervise* practice change initiatives or implementation/ quality improvement projects
- *Lead* practice change initiatives or implementation/ quality improvement projects
- *Participate in* practice change initiatives or implementation/ quality improvement projects
- None of the above – I do not supervise, lead, or participate in practice change initiatives or implementation/quality improvement projects

*If* ***Yes to 1 and*** ***any of the first three options in 2****, then the participant can move on to the rest of the questionnaire.*

*If* ***No to 1 or None of the above in 2****, then the participant will be told they are ineligible and prompted to close the questionnaire.*

**Part B: Demographics**

1. What is your gender identity?

*(Please check all that apply)*

- - Man
  - Woman
  - I identify my gender as (please specify): *[open text box to write answer]*
  - Prefer not to answer

1. What is your current role?

- Manager
- Program director
- Nurse
- Nurse educator
- Advanced practice nurse
- Midwife
- Physician
- Decision support
- Quality improvement or implementation support
- Other, please specify:__________________________

1. What is the highest level of education that you have completed?

- High school diploma
- College diploma
- Bachelor’s degree
- Master’s degree
- Doctoral degree
- Other:___________________

1. How many years of experience do you have in maternal-newborn care? ______________years
2. How many years of experience do you have *supervising or leading* practice change initiatives or implementation/quality improvement projects? ______________years

How many years of experience do you have *participating in* implementation projects, quality improvement projects, or practice change initiatives? ______________years

1. Have you had formal training (e.g., courses, workshops, programs) in any of the following areas? *(Please check all that apply)*

- Knowledge translation (KT), knowledge mobilization, implementation practice
- Implementation science
- Quality improvement

1. How knowledgeable are you about using evidence-informed approaches to change clinical practice?
   - Extremely knowledgeable
   - Very knowledgeable
   - Moderately knowledgeable
   - Slightly knowledgeable
   - Not at all knowledgeable
2. How confident are you in following an evidence-informed approach to changing clinical practice?
   - Extremely confident
   - Very confident
   - Moderately confident
   - Slightly confident
   - Not at all confident
3. What type of maternal-newborn unit(s) do you currently work in?

(Please check all that apply)

- Labour and delivery
- Postpartum
- Neonatal intensive care unit
- Special Care Nursery
- Other, please specify:

1. What level of **perinatal and birthing** care does your hospital provide? *[this question will appear if respondent selects labour and delivery or postpartum in question above].*

- Level I
- Level II
- Level III

1. What level of **newborn** care does your hospital provide? *[this question will appear if respondent selects SCN or NICU in question above].*

- Level I
- Level II
- Level III

1. What regional network is your hospital part of?

*(As per PCMCH: https://www.pcmch.on.ca/regional-networks-operational-forum/)*

- Maternal, Newborn, Child and Youth Network (MNCYN)
- Southern Ontario Maternal Child and Youth Network (SOMCYN)
- Women & Children’s Health Network (WCHN)
- Toronto Region Maternal Child Health Network (TRMCHN)
- Northern Maternal Child Network (NMCN)
- Champlain Maternal Newborn Regional Program (CMNRP) and Kids Come First Health Team (KCFHT)
- We are not currently part of a regional network
- Unsure

1. Which hospital do you work in?

*(Please note that this information will only be used to facilitate reminders about questionnaire completion and will not be reported)*

_______________________________________

A [recent study](https://www.cmaj.ca/content/194/8/E279) identified that about 30% of all care in Canada is “inappropriate,” meaning that patients have not received care that aligns with the best available evidence. This suggests that many practices (e.g., diagnostics, therapies and procedures, medications) are being overused (i.e., used when not needed), underused (i.e. not used when needed), or misused (i.e., used incorrectly).

*As you answer the next question(s), think about your overall experiences and knowledge from working in maternal-newborn care.*

I think that the proportion of inappropriate care (overuse, underuse, misuse) in Ontario maternal-newborn care settings is:

Higher than the 30% average reported in the study

About the same as the 30% average reported in the study

Lower than the 30% average reported in the study

(If higher or lower than 30% average) In Ontario maternal-newborn hospital settings, what proportion of care do you think is "inappropriate"? (i.e., diagnostics, therapies, procedures, and medications that are overused, underused, or misused)

Change the slider to set a response.

|  | % |  |
| --- | --- | --- |
| 0 |  | 100 |

Please briefly state any clinical practice change initiatives your unit is *currently* working on (e.g., reducing caesarean sections, increasing breastfeeding rates, etc.)

|  |
| --- |

Please briefly state any clinical practice change initiatives your unit is *planning* to work on in the future.

|  |
| --- |

**Part C: Usual Approaches to Practice Change Initiatives^[[1]](#footnote-2)^**

Changing practice can involve many steps, and different teams may use different processes and steps to implement changes. In this next section, we present 28 different steps that you may or may not do. Please think about how your team *typically* approaches clinical practice changes in your setting. Based on your experience, we are interested in:

- which steps your team thinks are important or not;
- which steps your team typically does or not; and
- which steps your team feels confident doing or not.

Please note that the following questions focus on your *team*. In some cases, you may feel uncertain about how to answer questions about your team, but we ask that you do your best to pick the answer that reflects your perception of your team.

Throughout this section we use the term “practice change initiative” to refer to any formal attempt to change a clinical practice, care process, or policy in your hospital setting. A practice change initiative could be focused at the individual level (e.g., trying to change the way healthcare professionals provide care) or at a unit/organizational level (e.g., trying to implement a new process or policy), but the end goal is to improve an outcome.

We also use the term “stakeholders” to refer to individuals who are interested in and/or affected by the practice change initiative. Examples of stakeholders include pregnant and birthing people and their families, healthcare providers, and managers/leaders.

|  | A. How important is this step to your team? | | | B. How often does your team do this step? | | | C. How confident is your team doing this step? | | |
| --- | --- | --- | --- | --- | --- | --- | --- | --- | --- |
|  | **Very important** | **Somewhat important** | **Not at all important** | **Always** | **Sometimes** | **Never** | **Very confident** | **Somewhat confident** | **Not at all confident** |
| First, let’s think about identifying a problem that needs to be solved (e.g., inappropriately high or low rates of a clinical practice) and assembling a team to work on solving the problem. | | | | | | | | | |
| 1. Identify a problem or issue that is relevant to stakeholders (such as patients, families, staff) | ⭘ | ⭘ | ⭘ | ⭘ | ⭘ | ⭘ | ⭘ | ⭘ | ⭘ |
| 1. Form a working group/team with diverse expertise to work on the practice change initiative | ⭘ | ⭘ | ⭘ | ⭘ | ⭘ | ⭘ | ⭘ | ⭘ | ⭘ |
| 1. Involve stakeholders as partners throughout the practice change initiative | ⭘ | ⭘ | ⭘ | ⭘ | ⭘ | ⭘ | ⭘ | ⭘ | ⭘ |
| 1. Create a formal implementation plan for the practice change initiative | ⭘ | ⭘ | ⭘ | ⭘ | ⭘ | ⭘ | ⭘ | ⭘ | ⭘ |
| Next, let’s think about the potential “thing” to be implemented to solve the problem. The “thing” could be a program, a guideline, a guideline recommendation, a practice, or an innovation. | | | | | | | | | |
| 1. Use research evidence (e.g., research studies, systematic reviews, clinical practice guidelines) to identify potential programs, guidelines, practices, or innovations to solve the identified problem | ⭘ | ⭘ | ⭘ | ⭘ | ⭘ | ⭘ | ⭘ | ⭘ | ⭘ |
| 1. Assess the quality of the program, guideline, practice, or innovation (e.g., strength of evidence, applicability, up to date) | ⭘ | ⭘ | ⭘ | ⭘ | ⭘ | ⭘ | ⭘ | ⭘ | ⭘ |
| Next, let’s think about “current practice” (what people are doing in your setting) and how it is different from the “best practice” (i.e., what the program, guideline, practice, innovation recommends should be done). | | | | | | | | | |
| 1. Identify or create a tangible indicator of best practice (i.e., a measure to compare against to see if best practice is occurring in your setting) | ⭘ | ⭘ | ⭘ | ⭘ | ⭘ | ⭘ | ⭘ | ⭘ | ⭘ |
| 1. Collect local data to learn about current practice in your setting (e.g., from chart audits, administrative data, or patient and staff surveys) | ⭘ | ⭘ | ⭘ | ⭘ | ⭘ | ⭘ | ⭘ | ⭘ | ⭘ |
| 1. Compare current practice in your setting to the best practice to determine how big the “gap” (problem) is | ⭘ | ⭘ | ⭘ | ⭘ | ⭘ | ⭘ | ⭘ | ⭘ | ⭘ |
| Next, let’s think about the decision of what “best practice” (program, guideline, recommendation, practice, innovation) to implement. | | | | | | | | | |
| 1. Work as a team to select the best practice to be implemented | ⭘ | ⭘ | ⭘ | ⭘ | ⭘ | ⭘ | ⭘ | ⭘ | ⭘ |
| 1. Analyze the best practice in terms of who needs to do what, when, to whom, and under what circumstances | ⭘ | ⭘ | ⭘ | ⭘ | ⭘ | ⭘ | ⭘ | ⭘ | ⭘ |
| 1. Confirm that key stakeholders (e.g., staff, leaders, patients) endorse/support the selected best practice | ⭘ | ⭘ | ⭘ | ⭘ | ⭘ | ⭘ | ⭘ | ⭘ | ⭘ |
| 1. Customize the selected best practice (as needed) to make it work within your setting | ⭘ | ⭘ | ⭘ | ⭘ | ⭘ | ⭘ | ⭘ | ⭘ | ⭘ |
| Next, let’s think about the process for considering how easy or difficult it will be to implement the selected best practice (i.e., program, guideline, recommendation, practice, or innovation). | | | | | | | | | |
| 1. Conduct a stakeholder analysis (i.e., mapping key stakeholders and their level of influence and support) | ⭘ | ⭘ | ⭘ | ⭘ | ⭘ | ⭘ | ⭘ | ⭘ | ⭘ |
| 1. Systematically assess the potential barriers (challenges) and drivers (facilitators) to implementing the selected best practice | ⭘ | ⭘ | ⭘ | ⭘ | ⭘ | ⭘ | ⭘ | ⭘ | ⭘ |
| 1. Prioritize the identified barriers (challenges) that that are feasible to address to successfully implement the selected best practice | ⭘ | ⭘ | ⭘ | ⭘ | ⭘ | ⭘ | ⭘ | ⭘ | ⭘ |
| Next, let’s think about *how* the “best practice” (program, guideline, recommendation, practice, or innovation) is actually implemented in your setting. Think about the “change strategies” that may be used to promote or encourage use of the new program, guideline, practice, or innovation. These “change strategies” could include educational strategies, audit and feedback, or incentives, for example. | | | | | | | | | |
| 1. Select appropriate change strategies to specifically address the identified barriers (i.e., selecting an educational strategy after identifying that lack of knowledge is a barrier) | ⭘ | ⭘ | ⭘ | ⭘ | ⭘ | ⭘ | ⭘ | ⭘ | ⭘ |
| 1. Field-test (or pilot) the selected change strategies | ⭘ | ⭘ | ⭘ | ⭘ | ⭘ | ⭘ | ⭘ | ⭘ | ⭘ |
| 1. Complete a pre-launch checklist before fully implementing the best practice | ⭘ | ⭘ | ⭘ | ⭘ | ⭘ | ⭘ | ⭘ | ⭘ | ⭘ |
| Next, let’s think about *planning* for monitoring, evaluating, and sustainability. By “planning” we mean establishing what, when, and how your practice change initiative will be monitored, evaluated, and sustained. | | | | | | | | | |
| 1. Create a plan for a “process evaluation” to monitor the uptake of the best practice (i.e., are people doing the best practice?) | ⭘ | ⭘ | ⭘ | ⭘ | ⭘ | ⭘ | ⭘ | ⭘ | ⭘ |
| 1. Create a plan for an “outcome evaluation” to determine if use of the selected best practice results in the desired *outcomes* (e.g., health outcomes) | ⭘ | ⭘ | ⭘ | ⭘ | ⭘ | ⭘ | ⭘ | ⭘ | ⭘ |
| 1. Create a “sustainability plan” outlining the actions the team will take to maintain use of the best practice over time | ⭘ | ⭘ | ⭘ | ⭘ | ⭘ | ⭘ | ⭘ | ⭘ | ⭘ |
| Now let’s think about *actioning* the plans for monitoring, evaluation, and sustainability. | | | | | | | | | |
| 1. Collect and analyze data to assess whether the best practice is being used (i.e., monitor the change *process)* | ⭘ | ⭘ | ⭘ | ⭘ | ⭘ | ⭘ | ⭘ | ⭘ | ⭘ |
| 1. Collect and analyze data to assess whether use of the best practice resulted in the desired outcomes (i.e., evaluate the *outcomes and impact*) | ⭘ | ⭘ | ⭘ | ⭘ | ⭘ | ⭘ | ⭘ | ⭘ | ⭘ |
| 1. Use the monitoring and evaluation findings to *adjust the change strategies* as needed | ⭘ | ⭘ | ⭘ | ⭘ | ⭘ | ⭘ | ⭘ | ⭘ | ⭘ |
| 1. Use strategies to help sustain use of the best practice over time | ⭘ | ⭘ | ⭘ | ⭘ | ⭘ | ⭘ | ⭘ | ⭘ | ⭘ |
| 1. Collect and analyze data to assess whether the sustainability strategies are helping to maintain use of the best practice (i.e., monitor sustainability strategies) | ⭘ | ⭘ | ⭘ | ⭘ | ⭘ | ⭘ | ⭘ | ⭘ | ⭘ |
| Finally, let’s think about equity, diversity, and inclusion. | | | | | | | | | |
| 1. Consider equity, diversity, and inclusion (EDI) in the practice change process | ⭘ | ⭘ | ⭘ | ⭘ | ⭘ | ⭘ | ⭘ | ⭘ | ⭘ |

1. You indicated that your team “sometimes” or “always” considers equity, diversity, and inclusion (EDI) in the practice change process. During which steps does your team explicitly consider EDI?

- When creating the core working group *(e.g., aiming for diversity and representation on the team)*
- When consulting with interested and affected parties *(e.g., aiming for consultation with a diverse group)*
- When prioritizing what practice problem needs to be addressed *(e.g., prioritizing problems to improve equity)*
- When selecting or modifying the specific solution (guideline, program, practice) to be implemented *(e.g., using solutions that meet EDI criteria)*
- When learning about potential barriers to implementing the solution *(e.g., including questions to explore EDI considerations)*
- When evaluating the practice change *(e.g., selecting EDI-relevant outcome measures)*
- When working to sustain the practice change *(e.g., including EDI considerations for sustaining the change)*
- Other (specify):__________________________________________________________________

1. Who is usually *primarily responsible* *for* *leading* the practice change process in your setting (i.e., the person leading the implementation team)?

**The people leading practice changes may be different depending on the type of change. For this question, think about what happens most often.*

- Program director
- Manager
- Clinical educator
- Nurses working at point-of-care
- Physicians
- Midwives
- Decision support personnel/analysts
- Pregnant and birthing people
- Family members/caregivers
- Other:_________________________________
- Unsure

Please enter any comments you have on who typically leads the practice change process in your setting: __________________________________________________________________________________________________________________________________________________________________________

1. Who typically contributes to the different phases of the practice change process in your setting?

**The people involved in practice changes may be different depending on the type of change. For this question, think about what happens most often.*

|  | *Identifying a problem* that needs to be addressed | *Developing solution(s)* to address the problem | *Implementing* the *solution(s)* in practice |
| --- | --- | --- | --- |
| Program director | 🞎 | 🞎 | 🞎 |
| Manager | 🞎 | 🞎 | 🞎 |
| Clinical educators | 🞎 | 🞎 | 🞎 |
| Nurses working at point-of-care | 🞎 | 🞎 | 🞎 |
| Physicians | 🞎 | 🞎 | 🞎 |
| Midwives | 🞎 | 🞎 | 🞎 |
| Decision support personnel/analysts | 🞎 | 🞎 | 🞎 |
| Pregnant and birthing people | 🞎 | 🞎 | 🞎 |
| Family members/caregivers | 🞎 | 🞎 | 🞎 |
| Other (specify): | 🞎 | 🞎 | 🞎 |
| Unknown | 🞎 | 🞎 | 🞎 |

Please enter any other comments you have on who contributes to the different phases in the practice change process in your setting: ____________________________________________________________________________________________________________________________________________________________________

1. Do you currently use any of the following systems to engage people in the practice change process?

- Staff unit councils
- Patient and Family Advisory Councils
- Ticket systems for *staff* to submit ideas and feedback
- Ticket systems for *patients and families* to submit ideas and feedback
- Staff huddles
- Unsure
- Other:____________________________

1. Does your organization have a formal process that is used to facilitate clinical practice change initiatives?
   - Yes, we have a *mandatory* process that must be used for clinical practice changes
   - Yes, we have an *optional* process that we may choose to use (or not) for clinical practice changes
   - No, we do not have a process
   - Unsure

33a) If yes to Question 33, please briefly describe process:__________________________________

33b) If yes to Question 33, does your team typically use a formal framework/model/theory to facilitate the clinical practice change process?

- Yes
- No
- Unsure

33c) If yes to Question 33b, please indicate which framework(s) you use to guide the clinical practice change process?

*(Please check all that apply)*

- Plan, Do, Study, Act (PDSA)
- Root Cause Analysis
- SWOT Analysis
- LEAN Methodology
- Knowledge to Action Framework
- Other framework, please specify:_____________________________________________

If no or unsure to Question 33, do you think having a formal process to guide clinical practice change initiatives would be helpful to your team?

- Yes
- No
- Maybe
- Unsure

1. Other comments about the clinical practice change process typically used in your setting:______________________________________________________________________________________________________________________________________________________________
2. To what extent do you agree with the following statements?

|  | **Strongly agree** | **Agree** | **Neither agree nor disagree** | **Disagree** | **Strongly disagree** |
| --- | --- | --- | --- | --- | --- |
| Our team could benefit from *increasing our knowledge* about evidence-informed approaches to implementing clinical practice changes | ⭘ | ⭘ | ⭘ | ⭘ | ⭘ |
| Our team could benefit from *increasing our skills* to apply evidence-informed approaches to implementing clinical practice changes | ⭘ | ⭘ | ⭘ | ⭘ | ⭘ |
| Our team could benefit from *having an evidence-informed process* to guide the clinical practice change process in our setting | ⭘ | ⭘ | ⭘ | ⭘ | ⭘ |
| Our team is motivated to *use evidence-informed processes and strategies* to bring about clinical practice changes | ⭘ | ⭘ | ⭘ | ⭘ | ⭘ |
| Our team would value (appreciate) a *resource or toolkit* to guide our clinical practice change process | ⭘ | ⭘ | ⭘ | ⭘ | ⭘ |

**Part D: Resources to Support Practice Change Initiatives**

We are currently developing an evidence-informed toolkit (e.g. a collection of information and resources) to guide maternal-newborn teams through the process of implementing clinical practice changes. The next questions are about this new resource.

1. Generally, how does your team prefer to learn new information? From the list below, please select your **top three** learning approaches.
   - Independent, self-directed learning using educational materials
   - Online courses or workshops that are directed by an instructor
   - In-person courses or workshops that are directed by an instructor
   - Learning by doing (experiential learning)
   - Tailored coaching or mentoring by a content expert
   - Participating in a community of practice to learn from others
   - Other, please specify:_____________________________________________________
2. How important would the following features be in a practice change toolkit?

|  | **Very important** | **Somewhat important** | **Not important** |
| --- | --- | --- | --- |
| Information explaining *what* steps to take in the clinical practice change process | ⭘ | ⭘ | ⭘ |
| Information explaining *why* undertaking various steps in the clinical practice change process is critical | ⭘ | ⭘ | ⭘ |
| Information explaining *how* to undertake various steps in the clinical practice change process | ⭘ | ⭘ | ⭘ |
| *Lessons learned* from other maternal-newborn teams during their practice change initiatives | ⭘ | ⭘ | ⭘ |
| *Links to other resources* to support practice change initiatives | ⭘ | ⭘ | ⭘ |
| *Tools and templates* to support practice change initiatives | ⭘ | ⭘ | ⭘ |
| Access to an *online version* of the toolkit | ⭘ | ⭘ | ⭘ |
| Access to a *paper-based version* of the toolkit | ⭘ | ⭘ | ⭘ |

1. Are there any other important features that you would like in a practice change toolkit?_____________________________________________________________________________________________________________________________________________________________
2. When the toolkit becomes available, how likely would your team be to use it?

- Very likely
- Likely
- Neutral
- Unlikely
- Very unlikely
- I don’t know

1. When the toolkit becomes available, would any of these additional resources be of interest to you and your team?

*(Please check all that apply)*

- Webinar providing an overview of the toolkit’s content and how to use it
- Ongoing support from a BORN staff member to advise on using the toolkit
- Community of practice to connect with other maternal-newborn teams using the toolkit
- Brief user tip sheets on how to apply the toolkit
- A webpage to access toolkit content, tools, and templates
- A slide deck on the toolkit’s content that can be tailored for your own use
- Other ideas, please specify:_________________________________________________

**Part E: Perceptions of BORN’s role**

1. If BORN Ontario offered training on how to apply the toolkit in your practice change initiatives, how likely would your team be to access this training?

- Very likely
- Likely
- Neutral
- Unlikely
- Very unlikely
- I don’t know

1. If BORN Ontario offered ongoing support and consultations on how to implement practice changes in maternal-newborn care, how likely would your team be to use this service?

- Very likely
- Likely
- Neutral
- Unlikely
- Very unlikely
- I don’t know

If answered “unlikely” or “very unlikely” to Question 35 and/or Question 36…

Please tell us why your team would be “unlikely” or “very unlikely” to use the training or support: *(Please check all that apply)*

- Our team does not require additional education or training on implementing practice change initiatives
- Our team has access to other *internal* resources to support practice change initiatives
- Our team has access to other *external* resources to support practice change initiatives
- BORN is not an organization that our team recognizes as having the expertise to support our practice change initiatives
- Other reasons, please specify:______________________________________________________

**Part F: Future contact**

In about 6-12 months we plan to have a draft of the toolkit ready for review. At that time, we will be looking for volunteers to read the toolkit draft and provide feedback on what they like, what they do not like, what is missing, and how the toolkit could be improved to best meet the needs of maternal-newborn teams.

1. Would you be willing to be contacted in 6-12 months to be asked if you might be interested in taking part in reviewing the toolkit?

*Please know that selecting “yes” does not commit you to taking part in future study phases. You will receive a study invitation with more information at a later date, and you can make a decision to take part or not at that time.*

- Yes
- No

**[If participant selects yes]:**

Thank you for your willingness to be contacted in the upcoming months for future phases of this study! Please click on the link below to enter in your details. The contact form will open in a new tab to ensure that your personal information is not associated with your questionnaire responses.

[URL]

*Info at new contact URL:*

Thank you for agreeing to be contacted by our project team in the future about reviewing the toolkit draft. Please know that providing your name and contact information here does not commit you to any future involvement. You will receive a study invitation with more information at a later date, and you can make a decision to take part or not at that time.

*Please note that because this information is not connected with your questionnaire, you may be asked to answer similar questions again. We appreciate you taking the time to enter this information.*

1. Please enter your email address to use for future contact:___________________________________
2. What type of unit do you work in?

*(Please check all that apply)*

- Labour and delivery
- Postpartum
- Neonatal Intensive Care
- Special Care Nursery
- Other, please specify:________________

1. What level of **perinatal and birthing** care does your hospital provide? *[this question will appear if respondent selects labour and delivery or postpartum in question above].*

- Level I
- Level II
- Level III

1. What level of **newborn** care does your hospital provide? *[this question will appear if respondent selects SCN or NICU in question above].*

- Level I
- Level II
- Level III

1. What is your role?

- Manager
- Program director
- Nurse
- Nurse educator
- Advanced practice nurse
- Midwife
- Physician
- Decision support
- Quality improvement or implementation support
- Other, please specify:__________________________

1. Please rate your level of experience in the following activities:

|  | Novice | Intermediate | Expert |
| --- | --- | --- | --- |
| Implementing practice change initiatives | ⭘ | ⭘ | ⭘ |
| Quality improvement projects | ⭘ | ⭘ | ⭘ |

1. Other comments:___________________________________________________________________

1. Note that in the online version of the questionnaire, this section was presented as a series of individual questions, rather than as a matrix. [↑](#footnote-ref-2)
