# Supplementary material for: Implementation processes and capacity-building needs in Ontario maternal-newborn care hospital settings: a cross-sectional survey
Source: BMC Nurs. 2025 Jan 6;24:10. doi: 10.1186/s12912-024-02643-z (PMC11702017; doi:10.1186/s12912-024-02643-z)
Supplement: Supplementary file 3 — Additional file 3. Organizational-level demographics. This file includes a table presenting characteristics of the respondents’ organizations, including a comparison to the province at large. [file 12912_2024_2643_MOESM3_ESM.docx]

**Additional file 3. Organizational-level demographics (N=57)^a^**

| **Variable** | **Survey sample**  **n (%)** | **Comparison to province (N=91)**  **n (%)** |
| --- | --- | --- |
| **Level of care – n (%)^b^** | |  |
| Low risk (level 1) | 24 (42.1) | 40 (44.0) |
| Moderate risk (level 2) | 25 (43.9) | 41 (45.0) |
| High risk (level 3) | 8 (14.0) | 10 (11.0) |
| **Geographical region – n (%)^b^** | |  |
| Central | 16 (28.1) | 22 (24.2) |
| East | 14 (24.6) | 20 (22.0) |
| North East | 7 (12.3) | 10 (11.0) |
| North West | 5 (8.8) | 6 (6.6) |
| Toronto | 4 (7.0) | 6 (6.6) |
| West | 11 (19.3) | 27 (29.7) |

^a^Across the 73 respondents, 57 organizations were represented: 12 hospitals had 2 responses; 1 hospital had 4 responses. 1 respondent did not indicate what hospital they were from.

^b^As per the Provincial Council for Maternal and Child Health (PCMCH) [25]
