# Supplementary material for: Implementation processes and capacity-building needs in Ontario maternal-newborn care hospital settings: a cross-sectional survey
Source: BMC Nurs. 2025 Jan 6;24:10. doi: 10.1186/s12912-024-02643-z (PMC11702017; doi:10.1186/s12912-024-02643-z)
Supplement: Supplementary file 4 — Additional file 4. Categories of practice change initiatives/implementation projects identified by survey respondents. This file includes a table presenting the different categories of practice change initiatives and implementation projects respondents indicated their organizations are currently or planning to work on. [file 12912_2024_2643_MOESM4_ESM.docx]

**Additional file 4. Categories of practice change initiatives/implementation projects identified by survey respondents (N=69)**

| **Category*** | **Number of respondents** | | **Example(s) of responses** |
| --- | --- | --- | --- |
|  | **Currently working on practice change in this area** | **Plan to work on practice change in this area** |  |
| Infant feeding | 20 | 5 | - Increasing breastfeeding rates - Becoming BFI designated |
| Inductions | 17 | 5 | - Reducing elective inductions of labour - Standardizing induction booking |
| Caesarean births | 14 | 2 | - Reducing caesarean birth rates - Improving caesarean birth scheduling |
| Oxytocin | 13 | 0 | - Implementing PCMCH Oxytocin Induction Recommendations |
| Education & training | 11 | 7 | - Bringing MORE^OB^ to our hospital - Sending staff to higher volume centers for experience |
| Internal service re-organization | 10 | 6 | - Implementing LDRP model of care - Implementing late preterm care on postpartum units |
| Postpartum hemorrhage | 8 | 3 | - PPH risk assessment and reduction |
| Fetal health surveillance | 8 | 0 | - Implementing newest SOGC Fetal Health Surveillance guideline |
| Infection prevention and treatment | 7 | 0 | - Decreasing nosocomial infection rates - Antibiotic stewardship |
| Triage practices | 7 | 1 | - Standardizing triage by implementing OTAS |
| Skin-to-skin care | 6 | 2 | - Increasing skin to skin for the first 4 hours of admission to NICU |
| Collaboration with external organizations & providers | 5 | 3 | - Increasing HBHC referrals |
| Pain management | 5 | 3 | - Initiating newborn pain management policy |
| Skin-to-skin care | 5 | 2 | - Increasing skin-to-skin care |
| VBAC/TOLAC | 4 | 2 | - Implementing TOLAC Bundle |
| Documentation and charting | 4 | 2 | - Developing standard electronic documentation |
| Patient experience and EDI | 4 | 1 | - EDI work in front facing care |
| Hyperbilirubinemia | 4 | 0 | - Introducing a hyperbilirubinemia procedure |
| Extremely premature infant care | 2 | 4 | - Improving shared decision making for extremely premature infants |
| Antenatal | 2 | 3 | - Developing Antepartum Standards of Practice |
| Neonatal Abstinence Syndrome | 2 | 3 | - Implementing Eat, Sleep, Console |

*All other topics were reported by 3 or less respondents. Examples include: admission practices (n=3); length of stay (n=3); patient education (n=3); blood draws (n=3); hypoglycemia (n=2); developmental care (n=2); mental health (n=2); plus 44 other topics listed by one respondent only.

*Abbreviations:* **BFI**: Baby Friendly Initiative; **EDI**: Equity, Diversity, and Inclusion; **HBHC**: Healthy Babies Healthy Children; **LDRP**: Labor, Delivery, Recovery, and Postpartum; **MORE^OB^**: Managing Obstetrical Risk Efficiently; **NICU**: Neonatal Intensive Care Unit; **OTAS**: Obstetric Triage Acuity Scale; **PCMCH**: Provincial Council for Maternal and Child Health; **PPH**: Postpartum Hemorrhage; **SOGC**: Society of Obstetricians and Gynaecologists of Canada; **TOLAC**: Trial Of Labor After Caesarean.
