# Supplementary material for: Implementation processes and capacity-building needs in Ontario maternal-newborn care hospital settings: a cross-sectional survey
Source: BMC Nurs. 2025 Jan 6;24:10. doi: 10.1186/s12912-024-02643-z (PMC11702017; doi:10.1186/s12912-024-02643-z)
Supplement: Supplementary file 5 — Additional file 5. Implementation activities done (reported by respondent). This file includes a figure presenting the responses on frequency of implementation activity completion (always, sometimes, never) for the 63 respondents for whom we had complete data on that series of questions. [file 12912_2024_2643_MOESM5_ESM.pdf]

Additional file 5. Implementation activities done (reported by respondent)

ID	Hospital ID	PHASE 1									PHASE 2											PHASE 3								# steps "Always" done
		1	2	3	4	5	6	7	8	9	10	11	12	13	14	15	16	17	18	19	20	21	22	23	24	25	26	27	28	
142	37	+	+	+	+	+	+	+	+	+	+	+	+	+	+	+	+	+	+	+	+	+	+	+	+	+	+	+	+	28
108	52	+	+	+	+	+	+	+	+	+	+	+	+	+	~	+	+	+	~	+	+	+	+	+	+	+	+	+	+	26
41	31A	+	+̲	+̲	+̲	+	+̲	+	+̲	+	+	+̲	+̲	+̲	+	+̲	+	+̲	~	+̲	+̲	+̲	+̲	+̲	+̲	~	+̲	+̲	+	26
84	10D	+	+̲	+	+̲	+̲	+	+̲	+̲	+̲	+	+	+̲	+	~̲	+̲	+̲	+̲	~̲	+̲	+̲	+̲	~̲	+̲	+̲	+̲	+	+̲	+̲	25
127	15	+	+	+	+	+	~	+	~	+	+	+	+	+	+	+	+	+	~	~	+	~	+	+	+	+	+	~	~	21
75	10A	+	~̲	+	+̲	+̲	+	+̲	+̲	+̲	+	+	+̲	+	+̲	+̲	+̲	+̲	~̲	~̲	+̲	~̲	~̲	~̲	+̲	+̲	+	+̲	~̲	21
78	10B	+	+̲	+	~̲	+̲	+	+̲	+̲	+̲	+	+	+̲	+	+̲	~̲	+̲	+̲	+̲	+̲	+̲	~̲	+̲	~̲	~̲	~̲	+	~̲	+̲	21
140	45	+	+	+	~	+	+	~	+	+	+	+	+	+	~	+	+	+	+	~	+	—	~	+	+	+	+	+	~	21
144	38	+	+	+	+	~	+	+	~	~	+	+	+	+	~	+	+	+	~	~	+	~	+	+	+	+	~	~	+	19
46	-	+	+	+	+	+	+	+	+	+	+	+	+	~	~	+	~	+	~	~	+	~	~	~	~	~	+	~	+	17
89	20	+	+	+	+	+	+	+	+	+	+	+	+	+	~	+	+	+	~	~	~	~	~	~	~	~	~	~	~	16
141	17	+	+	+	~	+	~	~	+	~	+	~	+	~	~	~	+	+	~	+	+	+	+	~	~	+	+	~	~	15
105	41	+	+	~	+	+	~	~	+	+	~	~	~	+	~	+	+	+	+	+	+	~	~	~	~	+	~	~	+	15
124	19	+	~	~	+	+	~	+	~	+	+	+	~	+	—	+	+	+	~	~	+	~	~	+	+	~	+	~	~	15
60	8	+	~	+	~	+	~	~	+	~	+	~	+	~	~	+	+	~	~	~	~	~	+	~	+	+	+	+	~	13
17	26	+	+	~	~	+	+	—	+	+	+	+	+	+	—	~	+	~	—	~	~	—	~	~	~	+	+	—	~	13
64	56	+	~	~	~	+	+	+	+	~	~	+	+	+	~	~	+	+	+	~	+	~	~	~	~	~	~	~	+	13
29	39A	+̲	~	~	+̲	+̲	+̲	+	+̲	+̳	~	+̲	~	~	~̳	~	+̲	~̲	~	~	~	+̲	~	~	+̲	~	~	~	+̲	12
67	48	+	~	~	+	+	+	+	~	+	~	~	~	~	~	~	~	~	~	~	+	~	+	+	+	+	~	~	+	12
101	54	+	~	~	~	+	+	~	~	+	~	+	~	~	~	~	~	+	+	~	+	+	+	+	~	~	~	~	~	11
131	33	+	~	~	~	~	~	~	+	+	~	~	~	~	~	+	~	+	+	+	+	—	~	~	+	+	~	~	~	10
76	44	+	+	+	~	~	+	~	~	~	+	+	~	+	~	~	+	+	+	~	~	~	~	—	~	~	~	~	~	10
5	27A	+̲	+̲	+̲	+̲	+̲	~	~̳	~	~	~	+̲	+̳	+̲	~̳	~	~	+̲	~	~̳	~̳	~̳	+̳	~	~	~	~	~	~	10
145	3	~	+	~	+	+	~	+	+	+	+	+	~	~	~	~	~	~	~	~	~	~	~	+	~	~	~	~	~	9
103	16	+	~	+	~	+	~	~	+	+	+	+	+	~	~	~	—	~	~	—	+	—	—	~	~	~	—	—	~	9
59	22	+	~	+	+	~	+	~	+	~	+	+	+	+	~	~	~	~	—	~	~	~	~	~	~	~	~	~	~	9
3	24	+	+	~	~	~	~	~	~	~	+	+	+	+	—	~	~	~	+	~	~	~	—	+	+	~	~	~	~	9
114	47	+	+	+	~	+	+	~	~	~	+	~	+	~	~	~	~	+	~	~	~	~	~	~	~	~	~	—	+	9
15	32B	~̲	+̲	~	+̳	+	+̲	~̳	~	+̲	~	+̲	~	+̲	~̳	~̳	~̳	+̲	~	~	+̲	~̳	~	~̳	~	~	~	~̳	~	9
125	12	+	~	~	+	+	~	~	+	~	+	~	+	~	~	~	~	~	~	+	~	~	~	~	~	~	+	~	~	8
106	50	~	+	~	~	+	+	~	~	+	~	~	+	+	~	~	~	~	~	~	~	~	~	+	~	~	~	~	+	8
81	10C	+	+̲	+	~̲	~̲	+	~̲	~̲	~̲	+	+	~̲	+	~̲	~̲	~̲	~̲	~̲	~̲	~̲	~̲	~̲	~̲	~̲	~̲	+	~̲	~̲	8
92	31B	+	~̲	~̲	~̲	+	~̲	+	~̲	+	+	~̲	~̲	~̲	+	~̲	+	~̲	~	~̲	~̲	~̲	~̲	~̲	~̲	~	~̲	~̲	+	8
107	46B	+	~	~	~̲	+	+	~	+̲	+̲	+̲	~	~	+	~	~	~	~̲	~	~	+̲	~̲	~	~	~	~	~	~̳	~̳	8

47	49B	~	~̲	+̲	~	+̲	+̲	+̲	~	+̲	~	+̲	+̲	~	~	~	~	~	+̲	~̳	~̳	~	~̳	~	~	~	~	~	~	8
88	40	~	~	~	~	+	+	+	~	+	~	~	~	+	~	~	+	~	~	~	~	~	~	~	~	~	~	~	+	7
71	46A	+	~	~	+̲	+	+	~	~̲	~̲	~̲	~	~	+	~	~	~	+̲	~	~	~̲	+̲	~	~	~	~	~	—̳	—̳	7
128	53A	~	+̲	~̲	+̲	~	~	~	+	+̲	+̲	~	+̲	~	—̳	~	~	~	~	~	~	~	~	~̲	~̲	~	~	~	+̳	7
16	21	+	~	+	~	~	~	~	+	~	~	+	~	~	+	~	~	+	~	~	~	~	~	~	~	~	~	—	~	6
83	2	+	~	~	~	+	+	+	+	~	~	~	~	~	—	—	—	~	~	~	~	~	~	~	~	~	~	~	~	5
129	4	+	~	~	+	+	~	~	~	~	~	~	~	+	~	~	~	+	~	~	~	~	~	~	~	~	~	~	~	5
21	11	~	~	+	~	~	~	—	+	~	+	~	~	~	+	+	~	~	—	—	—	—	—	—	—	~	—	—	~	5
45	13	+	~	+	~	+	+	~	~	~	~	~	~	~	~	~	~	~	~	~	~	—	~	~	~	~	~	~	+	5
19	14	~	~	~	~	~	~	—	~	~	~	~	+	~	~	~	~	~	+	+	~	~	~	~	+	+	~	~	~	5
33	36	+	~	~	~	~	~	~	+	~	+	+	~	~	—	~	~	~	~	~	~	~	~	~	~	~	~	~	+	5
27	43	+	~	+	~	+	~	~	~	~	~	~	+	+	—	~	~	~	~	—	~	—	~	~	—	—	—	—	~	5
136	5	~	~	—	~	+	~	~	~	~	+	~	+	+	—	~	~	~	~	—	~	—	—	—	—	—	~	—	~	4
95	34	+	+	~	~	+	~	~	+	~	~	~	~	~	~	~	~	—	~	~	~	~	—	~	~	~	~	~	~	4
56	35	+	~	~	—	—	—	—	~	~	+	~	~	+	—	~	~	~	~	~	~	—	~	—	—	~	~	—	+	4
150	53B	~	~̲	+̲	~̲	~	~	~	+	~̲	~̲	~	~̲	~	~̳	~	~	~	~	~	~	~	~	+̲	+̲	~	~	~	—̳	4
116	42	~	+	—	~	+	~	—	~	—	~	~	~	+	—	—	~	~	~	~	—	—	—	—	—	—	—	—	~	3
30	07B	+	~	~	~	~	~	~	~̳	~	~	~	~	~	~̳	~	~	~	~̳	+̲	~̳	~̳	~̳	~	~	~	~	~	+̲	3
39	23B	~	~	~	—̳	+̲	~	—	~	~	~	—̳	~	~	~	~	~	~̳	+̳	—̳	—	—	—̳	—̳	—̳	—	—̳	—̳	—	2
13	32A	+̲	~̲	~	—̳	+	~̲	—̳	~	~̲	~	~̲	~	~̲	—̳	—̳	—̳	~̲	~	~	~̲	—̳	~	—̳	~	~	~	—̳	~	2
143	39B	~̲	~	~	~̲	~̲	~̲	+	~̲	—̳	~	~̲	~	~	—̳	~	~̲	+̲	~	~	~	~̲	~	~	~̲	~	~	~	~̲	2
132	29	~	~	~	~	~	~	—	+	~	~	~	~	~	—	—	~	~	~	—	—	—	—	~	~	—	~	—	—	1
44	57	+	~	~	~	~	~	~	—	~	~	~	—	—	—	~	~	~	~	~	—	—	—	~	—	—	—	—	~	1
8	7A	+	~	~	~	~	~	~	—̳	~	~	~	~	~	—̳	~	~	~	—̳	—̳	—̳	—̳	—̳	~	~	~	~	~	~̲	1
34	49A	~	+̲	~̲	~	~̲	~̲	~̲	~	~̲	~	~̲	~̲	~	~	~	~	~	~̲	—̳	—̳	~	—̳	~	~	~	~	~	~	1
49	9	~	~	~	~	~	~	~	—	—	—	—	~	—	—	~	~	—	—	—	—	~	—	~	~	~	—	~	~	0
65	18	—	~	~	~	~	~	~	~	~	~	~	~	~	—	~	~	~	—	~	~	—	~	—	~	~	~	~	~	0
36	23A	~	~	~	~̳	~̲	~	—	~	~	~	~̳	~	~	~	~	~	—̳	—̲	~̳	—	—	~̳	~̳	~̳	—	~̳	~̳	—	0
130	27B	~̲	~̲	~̲	~̲	~̲	~	—̳	~	~	~	~̲	—̳	~̲	—̳	~	~	~̲	~	—̳	—̳	—̳	—̳	~	~	~	~	~	~	0

Legend

+ Respondent indicated their team "always" does this activity

~ Respondent indicated their team "sometimes" does this activity

— Respondent indicated their team "never" does this activity

— A single underline indicates a disagreement between two participants from the same site (always/sometimes)*

= A double underline indicates a disagreement between two participants from the same site (always/never or sometimes/never)*

**Notes:*

10 sites had more than one respondent (identified by the hospital ID number plus a letter). Any discrepancies between these respondents are indicated with a single or double underline (see legend above). When examining all disagreements, respondents disagreed on a median of 12.5 activities (range=8-21 activities). When excluding disagreements involving "always/sometimes", respondents disagreed on a median of 2.5 activities (range=0-10 steps).
