# Supplementary material for: Implementation processes and capacity-building needs in Ontario maternal-newborn care hospital settings: a cross-sectional survey
Source: BMC Nurs. 2025 Jan 6;24:10. doi: 10.1186/s12912-024-02643-z (PMC11702017; doi:10.1186/s12912-024-02643-z)
Supplement: Supplementary file 6 — Additional file 6. Importance, completion, and confidence with Implementation activities. This file includes a table presenting the descriptive statistics (frequency and percentages) for importance, completion, and confidence for each of the 28 implementation activities. [file 12912_2024_2643_MOESM6_ESM.docx]

**Additional file 6. Importance, completion, and confidence with implementation activities**

|  | | **How important is this step to your team?** | | | **How often does your team do this step?** | | | | **How confident is your team doing this step?^a^** | | |
| --- | --- | --- | --- | --- | --- | --- | --- | --- | --- | --- | --- |
|  |  | **Very important**  n (%) | **Somewhat important**  n (%) | **Not at all important**  n (%) | **Always**  n (%) | | **Sometimes**  n (%) | **Never**  n (%) | **Very confident**  n (%) | **Somewhat confident**  n (%) | **Not at all confident**  n (%) |
| **PHASE 1** | Identify a relevant problem or issue | 69 (94.5) | 4 (5.5) | 0 (0) | 49 (67.1) | | 23 (31.5) | 1 (1.4) | 33 (47.1)^b^ | 36 (51.4)^b^ | 1 (1.4)^b^ |
|  | Form a working group | 49 (67.1) | 23 (31.5) | 1 (1.4) | 28 (38.4) | | 45 (61.6) | 0 (0) | 26 (36.1)^c^ | 44 (61.1)^c^ | 2 (2.8)^c^ |
|  | Involve stakeholders as partners throughout change initiative | 56 (76.7) | 16 (21.9) | 1 (1.4) | 30 (41.1) | | 41 (56.2) | 2 (2.7) | 21 (30.0)^c^ | 48 (68.6)^c^ | 1 (1.4)^c^ |
|  | Create a formal implementation plan | 51 (69.9) | 20 (27.4) | 2 (2.7) | 23 (31.9)^c^ | | 46 (63.9)^c^ | 3 (4.2)^c^ | 17 (24.6)^c^ | 47 (68.1)^c^ | 5 (7.2)^c^ |
|  | Use research evidence to identify potential programs, guidelines, practices, or innovations to solve problem | 63 (86.3) | 9 (12.3) | 1 (1.4) | 45 (61.6) | | 27 (37.0) | 1 (1.4) | 42 (58.3) | 26 (36.1) | 4 (5.6) |
|  | Assess the quality of the program, guideline, practice, or innovation | 54 (74.0) | 17 (23.3) | 2 (2.7) | 29 (39.7) | | 43 (58.9) | 1 (1.4) | 25 (34.7) | 40 (55.6) | 7 (9.7) |
|  | Identify or create a tangible indicator of best practice | 48 (66.7)^c^ | 22 (30.6)^c^ | 2 (2.8)^c^ | 21 (29.2)^c^ | | 39 (54.2)^c^ | 12 (16.7)^c^ | 14 (23.7)^b^ | 39 (66.1)^b^ | 6 (10.2)^b^ |
|  | Collect local data to learn about current practice in your setting | 54 (74.0) | 19 (26.0) | 0 (0) | 30 (41.1) | | 40 (54.8) | 3 (4.1) | 26 (37.1) | 40 (57.1) | 4 (5.7) |
|  | Compare current practice in your setting to the best practice to determine how big the “gap” is | 49 (67.1) | 23 (31.5) | 1 (1.4) | 28 (38.4) | | 41 (56.2) | 4 (5.5) | 21 (30.9)^c^ | 42 (61.8)^c^ | 5 (7.4)^c^ |
| **PHASE 2** | Work as a team to select the best practice to be implemented | 62 (84.9) | 10 (13.7) | 1 (1.4) | 35 (47.9) | | 37 (50.7) | 1 (1.4) | 31 (43.1) | 39 (54.2) | 2 (2.8) |
|  | Analyze best practice for who needs to do what, when, to whom, and under what circumstances | 55 (75.3) | 16 (21.9) | 2 (2.7) | 29 (39.7) | | 42 (57.5) | 2 (2.7) | 20 (28.6)^c^ | 48 (68.6)^c^ | 2 (2.9)^c^ |
|  | Confirm that key stakeholders endorse the selected best practice | 59 (80.8) | 14 (19.2) | 0 (0) | 31 (42.5) | | 39 (53.4) | 3 (4.1) | 24 (34.3) | 43 (61.4) | 3 (4.3) |
|  | Customize the selected best practice for your setting | 57 (78.1) | 16 (21.9) | 0 (0) | 33 (45.2) | | 37 (50.7) | 3 (4.1) | 25 (36.2)^c^ | 42 (60.9)^c^ | 2 (2.9)^c^ |
|  | Conduct a stakeholder analysis | 34 (47.2)^c^ | 33 (45.8)^c^ | 5 (6.9)^c^ | 9 (12.5)^c^ | | 43 (59.7)^c^ | 20 (27.8)^c^ | 8 (15.4)^c^ | 38 (73.1)^c^ | 6 (11.5)^c^ |
|  | Assess the barriers and drivers to implementing the best practice | 46 (64.8)^b^ | 25 (35.2)^b^ | 0 (0)^b^ | 17 (23.6)^c^ | | 50 (69.4)^c^ | 5 (6.9)^c^ | 12 (17.9)^c^ | 48 (71.6)^c^ | 7 (10.4)^c^ |
|  | Prioritize the identified barriers that are feasible to address | 50 (68.5) | 22 (30.1) | 1 (1.4) | 21 (28.8) | | 47 (64.4) | 5 (6.8) | 18 (26.5) | 44 (64.7) | 6 (8.8) |
|  | Select change strategies to address the identified barriers | 56 (76.7) | 17 (23.3) | 0 (0) | 30 (41.7)^c^ | | 38 (52.8)^c^ | 4 (5.6)^c^ | 22 (32.4)^c^ | 43 (63.2)^c^ | 3 (4.4)^c^ |
|  | Field-test the selected change strategies | 32 (43.8) | 38 (52.1) | 3 (4.1) | 13 (17.8) | | 52 (71.2) | 8 (11.0) | 13 (20.0) | 50 (76.9) | 2 (3.1) |
|  | Create a plan for a “process evaluation” | 42 (59.2)^c^ | 26 (36.6)^c^ | 3 (4.2)^c^ | 12 (16.9)^b^ | | 47 (66.2)^b^ | 12 (16.9)^b^ | 10 (16.9)^b^ | 44 (74.6)^b^ | 5 (8.5)^b^ |
|  | Create a plan for an “outcome evaluation” | 46 (63.9)^c^ | 23 (31.9)^c^ | 3 (4.2)^c^ | 21 (29.2)^c^ | | 39 (54.2)^c^ | 12 (16.7)^c^ | 16 (27.1)^b^ | 37 (62.7)^b^ | 6 (10.2)^b^ |
| **PHASE 3** | Complete a pre-launch checklist | 23 (31.5) | 43 (58.9) | 7 (9.6) | 8 (11.0) | | 46 (63.0) | 19 (26.0) | 8 (14.8) | 46 (85.2) | 0 (0) |
|  | Create a “sustainability plan” | 45 (62.5)^c^ | 25 (34.7)^c^ | 2 (2.8)^c^ | 13 (18.1)^c^ | | 43 (59.7)^c^ | 16 (22.2)^c^ | 10 (17.9)^c^ | 41 (73.2)^c^ | 5 (8.9)^c^ |
|  | Use data to assess if the best practice is being used | 50 (70.4)^b^ | 18 (25.4)^b^ | 3 (4.2)^b^ | 15 (20.8)^c^ | | 49 (68.1)^c^ | 8 (11.1)^c^ | 16 (25.0)^c^ | 44 (68.8)^c^ | 4 (6.3)^c^ |
|  | Use data to assess if use of best practice resulted in the desired outcomes | 46 (63.9)^c^ | 24 (33.3)^c^ | 2 (2.8)^c^ | 17 (23.6)^c^ | | 46 (63.9)^c^ | 9 (12.5)^c^ | 15 (24.2)^b^ | 41 (66.1)^b^ | 6 (9.7)^b^ |
|  | Use the monitoring and evaluation findings to adjust the change strategies | 44 (62.0)^b^ | 26 (36.6)^b^ | 1 (1.4)^b^ | 15 (21.1)^b^ | | 48 (67.6)^b^ | 8 (11.3)^b^ | 14 (22.2)^b^ | 45 (71.4)^b^ | 4 (6.3)^b^ |
|  | Use strategies to sustain use of the best practice over time | 52 (73.2)^b^ | 18 (25.4)^b^ | 1 (1.4)^b^ | 16 (22.5)^b^ | | 47 (66.2)^b^ | 8 (11.3)^b^ | 12 (19.0)^b^ | 50 (79.4)^b^ | 1 (1.6)^b^ |
|  | Use data to assess if sustainability strategies are maintaining use of best practice | 34 (47.9)^b^ | 31 (43.7)^b^ | 6 (8.5)^b^ | 7 (9.7)^c^ | | 47 (65.3)^c^ | 18 (25.0)^c^ | 11 (20.4)^c^ | 40 (74.1)^c^ | 3 (5.6)^c^ |
|  | Consider equity, diversity, and inclusion (EDI) throughout the implementation process | 50 (71.4)^d^ | 20 (28.6)^d^ | 0 (0)^d^ | 23 (32.9)^d^ | | 41 (58.6)^d^ | 6 (8.6)^d^ | 16 (25.0)^d^ | 40 (62.5)^d^ | 8 (12.5)^d^ |
| ^a^Denominator only includes those who indicated they “always” or “sometimes” do the step.  ^b^Two respondents skipped this question; denominator adjusted accordingly.  ^c^One respondent skipped this question; denominator adjusted accordingly.  ^d^Three respondents skipped this question; denominator adjusted accordingly.  Note: 10 records had at least one missing value in this set of questions (63 had complete response set). | | | | | | **Green font** = Top quartile  **Red font** = Bottom quartile | | | | | |
