# Supplementary material for: Implementation processes and capacity-building needs in Ontario maternal-newborn care hospital settings: a cross-sectional survey
Source: BMC Nurs. 2025 Jan 6;24:10. doi: 10.1186/s12912-024-02643-z (PMC11702017; doi:10.1186/s12912-024-02643-z)
Supplement: Supplementary file 7 — Additional file 7. Equity, diversity, and inclusion (EDI) considerations during implementation process. This file includes a table presenting data on how respondents indicated they integrated equity, diversity, and inclusion (EDI) considerations into the implementation process. [file 12912_2024_2643_MOESM7_ESM.docx]

**Additional file 7. Equity, diversity, and inclusion (EDI) considerations during implementation process (N=64)**

| **Steps during which team explicitly considers EDI^a^** | **n (%)^b^** |
| --- | --- |
| When creating the core working group (e.g., aiming for diversity and representation on the team) | 41 (64.1) |
| When consulting with interested and affected parties (e.g., aiming for consultation with a diverse group) | 46 (71.9) |
| When prioritizing what practice problem needs to be addressed (e.g., prioritizing problems to improve equity) | 39 (60.9) |
| When selecting or modifying the specific solution (guideline, program, practice) to be implemented (e.g., using solutions that meet EDI criteria) | 32 (50.0) |
| When learning about potential barriers to implementing the solution (e.g., including questions to explore EDI considerations) | 44 (68.8) |
| When evaluating the practice change (e.g., selecting EDI-relevant outcome measures) | 22 (34.4) |
| When working to sustain the practice change (e.g., including EDI considerations for sustaining the change) | 24 (37.5) |

^a^Respondents could select more than one response option

^b^Denominator only includes those who answered “always” or “sometimes” to the question “How often does your team consider equity, diversity, and inclusion (EDI) throughout the implementation process?”
