# Supplementary material for: Implementation processes and capacity-building needs in Ontario maternal-newborn care hospital settings: a cross-sectional survey
Source: BMC Nurs. 2025 Jan 6;24:10. doi: 10.1186/s12912-024-02643-z (PMC11702017; doi:10.1186/s12912-024-02643-z)
Supplement: Supplementary file 8 — Additional file 8. Training, knowledge, and confidence related to using an evidence-informed approach to implement clinical practice changes. This file includes a table with the cross tabulation of respondents’ self-rated knowledge and confidence by whether they had previous training in of quality improvement, knowledge translation, implementation practice, or implementation science. [file 12912_2024_2643_MOESM8_ESM.docx]

**Additional file 8. Training, knowledge, and confidence related to using an evidence-informed approach to implement clinical practice changes (N=72)**

|  | | **Any previous training^a^** | **No previous training^a^** |
| --- | --- | --- | --- |
| Self-rated **knowledge** using evidence-informed approaches to change clinical practice | Extremely or very knowledgeable | 21 (43.8) | 4 (16.7) |
|  | Moderately knowledgeable | 21 (43.8) | 13 (54.2) |
|  | Not at all or slightly knowledgeable | 6 (12.5) | 7 (29.2) |
| Self-rated **confidence** using evidence-informed approaches to change clinical practice | Extremely or very confident | 26 (54.2) | 7 (28.0) |
|  | Moderately confident | 20 (41.7) | 13 (52.0) |
|  | Not at all or slightly confident | 2 (4.2) | 5 (20.0) |

^a^Training includes any one of quality improvement, knowledge translation, implementation practice, or implementation science.
